# A Dual Model for Prioritizing Cancer Mutations in the Non-coding Genome Based on Germline and Somatic Events

**DOI:** 10.1371/journal.pcbi.1004583

**Published:** 2015-11-20

**Authors:** Jia Li, Marie-Anne Poursat, Damien Drubay, Arnaud Motz, Zohra Saci, Antonin Morillon, Stefan Michiels, Daniel Gautheret

**Affiliations:** 1 Institute for Integrative Biology of the Cell, CNRS, CEA, Université Paris-Sud, Gif-sur-Yvette, France; 2 Laboratoire de Mathématique, Université Paris-Sud, Paris, France; 3 Service de Biostatistique et d’Epidémiologie, Gustave Roussy, Villejuif, France; 4 INSERM U1018, CESP, Université Paris-Sud, Villejuif, France; 5 RNA, epigenetics and genome fluidity, Institut Curie, PSL Research University, CNRS UMR3244, Université Pierre et Marie Curie, Paris, France; Johns Hopkins University, UNITED STATES

## Abstract

We address here the issue of prioritizing non-coding mutations in the tumoral genome. To this aim, we created two independent computational models. The first (germline) model estimates purifying selection based on population SNP data. The second (somatic) model estimates tumor mutation density based on whole genome tumor sequencing. We show that each model reflects a different set of constraints acting either on the normal or tumor genome, and we identify the specific genome features that most contribute to these constraints. Importantly, we show that the somatic mutation model carries independent functional information that can be used to narrow down the non-coding regions that may be relevant to cancer progression. On this basis, we identify positions in non-coding RNAs and the non-coding parts of mRNAs that are both under purifying selection in the germline and protected from mutation in tumors, thus introducing a new strategy for future detection of cancer driver elements in the expressed non-coding genome.

## Author Summary

Cancer cells undergo a mutation/selection process that resembles that of any living cell. Most mutations in cancer cell DNA occur in the so-called "non-coding" regions that represent 98.5% of the genome length. Pinning down which of these mutations contribute to the fitness of cancer cells would be important for identifying new "cancer drivers", which may in turn lead to future treatments. Unfortunately, predicting the impact of a non-coding DNA alteration remains extremely difficult. In this study, we analyze millions of non-coding cancer mutations and show cancer-specific mutational patterns can be used to predict non-coding regions that are preserved from mutations and may thus be important for cancer cell survival. Combining this information with population data, we propose a new scoring system that should help prioritize important non-coding mutations in future studies.

## Introduction

Since the onset of cancer genomics, the search for cancer genes and cancer-causing mutations has largely focused on protein-coding genes and, more specifically, their coding exons, where the damaging effect of mutations is best understood. Among 572 human genes considered as cancer drivers [1,2], nearly all are protein-coding. However protein-coding regions only represent a tiny subset of the vast transcribed area composed of over 50,000 non-coding genes [3,4] and the introns and untranslated regions (UTRs) of mRNA genes. Even though a large part of the non-coding transcribed regions is probably non functional [5], analyses based on evolutionary conservation or allele frequencies in human populations [6,7] estimate that 10 to 15% of the overall genome is under selection, that is 7–10 times larger than protein-coding regions.

Non-coding mutations may cause damage in many distinct ways. They may alter RNA structure [8] or binding sites for proteins or other RNAs, such as splicing sites [9] and microRNA target sites in 3’ UTRs, or impact regulatory sequences in gene promoters and enhancers. A recent population genomics study estimates that there are in average 15 highly deleterious mutations in the non-coding DNA of any healthy individual [10]. This large source of potentially damaging mutation remains mostly untouched by cancer genomics. In-depth analysis of the mutational load in the non-coding fraction of the genome is needed for the comprehensive understanding of cancer progression, as well as for the identification of new cancer drivers and therapeutic targets.

Whole genome normal *vs*. tumor sequencing commonly reveals thousands to tens of thousands of somatic mutations [11–13], scattered across all genomic areas. In coding regions the genetic code and aminoacid conservation rules provide a robust functional model for scoring mutational damage [14,15]. Similarly reliable tools are needed for non-coding regions in order to prioritize non-coding mutations and seek gene regions acquiring deleterious mutations at an unusual pace across a set of tumor samples. Several scoring systems for non-coding mutations already exist. The RegulomeDB system [16] scores variants using an empirical metric based on their overlap with transcription factor (TF) motifs, known TF binding site, chromatin marks or expression QTLs (eQTL) and thus is clearly centered on regulatory DNA variants. Other scoring models consider allele frequencies in human populations. Rare alleles are more often associated to reduced or lost gene activity than frequent alleles [17] and a high local ratio of rare to total SNP is indicative of purifying selection [10,18–20]. Khurana et al. used SNP data from the 1000 Genome project [21] to identify about 0,4% of the genome (12Mb) as sensitive to mutations and introduced an empirical scoring system (Funseq) to rate somatic mutations based on their presence in sensitive segments and overlap with known regulatory elements [10,22]. Likewise, the CADD system [23] predicts the deleteriousness of non-coding mutations based on allele frequencies modeled using machine learning on a series of genome features. Recently, Ritchie et al. introduced a model for prioritizing non-coding variants based on databases of known disease-related mutations [24]. The authors used machine learning to predict regions where disease-causing variants are most likely, using as explanatory variables functional features such as exon annotations, histone and other chromatin marks or transcription factor binding sites (TFBS). However useful, these models have limitations in that they are often directed towards the detection of regulatory elements (where 75% of disease variants have been located to date [24] and they only consider human mutations in the light of germline, evolutionary selection, meaning independently of a specific tissue or disease context. This latter point is especially important in cancer, where (1) most disease-inducing mutations occur somatically during the lifetime of an individual, and (2) these mutations may have different impacts when occurring in different tissues.

The availability of multiple whole genome sequence (WGS) data from tumors and matched normal tissue has revealed the extensiveness and singularity of cancer somatic mutations [11–13]. Cancer cells divide under their own set of selective constraints by which large regions of the genome can sustain high mutation rates while others seem relatively protected. This accelerated mutation rate is an important factor that may cause recurrent mutations in genome areas that are not necessarily related to cancer. Methods for scoring putative driver mutations now take such effect into account [13].

Variation of the somatic mutation rates in different genome areas is by itself a rich source of functional information. Schuster-Böckler & Lehner [25] related 45 functional features (mostly histone marks) to somatic mutation rates and observed that the major factor influencing mutation density was chromatin organization, marks of open chromatin being associated to a reduced SNV densities and marks of closed chromatin to higher densities. Cancer somatic mutations do not all cause cell death or tumor progression, but they may contribute to tumor heterogeneity which in turn facilitates the emergence of new clones capable of surviving micro-environmental changes and drug treatments [26]. In this sense, the somatic mutation landscape can be considered as a model of accelerated evolution in which most mutations are neutral and a handful is under selection as beneficial to tumor progression.

A strong hypothesis guiding the present study is that, in order to prioritize non-coding mutations in cancer and eventually discover new cancer drivers, one should take into account these dual selection forces acting on the tumor genome: (1) population and evolutionary constraints acting at germline level and (2) constraints resulting from the accelerated mutation background of the cancer tissue. To this aim we developed two integrative models that use annotated genome features to predict germline or somatic mutation constraints at any genomic location. We compared the functional features that most influence each mutational regimen and analyzed the intersection of constrained regions predicted under each model. A new picture of the somatic mutational landscape emerges where regions under constraint in the germline may be subject to highly variable mutation rates in the tumor. We present evidence that low somatic mutation areas are functionally relevant and can be used as a powerful screen for prioritizing cancer-related non-coding mutations.

## Results

We represent germline and somatic constraints acting on tumor genomes using two independent models, one for each mutational regimen, that we term the SNP model and the SOM model. For each model, we define a set of genome features, mainly from UCSC/Ensembl genome annotation and the ENCODE Project [27] and we use these features to predict the expected mutational constraint at any genome position. In the SNP model, the mutational constraint is expressed as a regional ratio of rare SNP, while in the SOM model it is expressed as a regional mutation density. We further describe each model below.

### Scoring mutations with the germline (SNP) model

**Fig 1:**
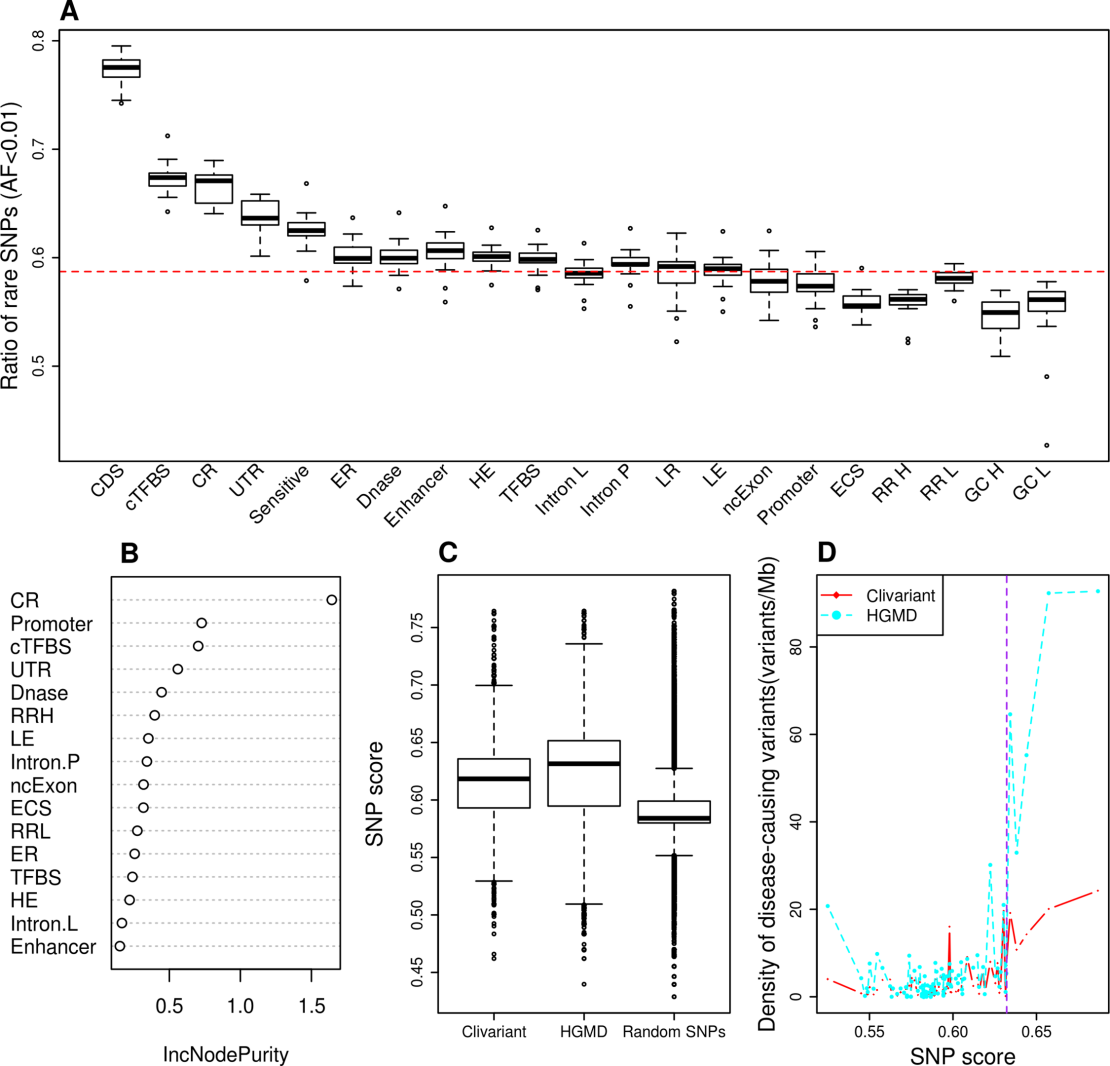
Construction of the rare SNP model. **A.** Fraction of rare SNPs (allele frequency <0.01) according to different genome features (see S1 Table and Methods for feature details). Each box shows rare SNP fraction across all human chromosomes, except chr. Y. CDS: coding sequence; cTFBS: conserved transcription factor binding site; CR: evolutionarily conserved region; UTR: untranslated region; Sensitive: region with high rate of rare SNP defined in [10], ER/LR: early and late replicated region; DNase: DNase I hypersensitive site; HE/LE: high and low expressed region; Intron L/Intron P: intron of lncRNA/of protein coding gene; ncExon: non coding exon; ECS: evolutionarily conserved structure; RR H/RR L/GC H/GC L: high recombination rate, low recombination rate, high GC content and low GC content regions. The red dotted line represents the average fraction of rare SNPs across the genome. **B.** Feature importance as measured by IncNodePurity. We only show here features that passed feature selection. **C**. Distribution of SNP scores for random SNPs and for clinical variants from the Clivariants and HGMD databases. Random SNPs here are a set of 1M random intergenic SNPs from the 1000 Genome project. **D**. Correlation of SNP scores with densities of disease-causing variants. Genome positions were sorted by SNP score and split into 20 Mb intervals. The plots show the average SNP score and density of disease-causing variants for each interval. The purple dotted line shows cutoff used for defining high SNP score thereafter.

A high regional ratio of rare SNPs (*i*.*e*. SNPs with allele frequencies below 0.5 or 1%) is a hallmark of genome regions under negative / purifying selection [10,18,20]. Fig 1A shows varying ratios of rare SNPs obtained from the 1000 Genome Project [21] associated to known functional regions or "features‴ (see S1 Table for each feature definition). Coding regions (CDS) clearly stand out as more constrained than non-coding regions in general. However, a number of non-coding elements also depart from the average genome signal, reflecting prior analysis of the 1000 Genome project data [10]. Regions under purifying selection (*ie*. with high rare SNP ratio) include evolutionarily conserved regions, transcription factor binding sites, DNase I hypersensitive sites, early replicated and highly expressed regions. Inversely, we observed low ratios of rare SNPs in regions of strong GC-bias, high replication rate and evolutionary conserved RNA structures (ECS). Of note, this low ratio of rare SNP in ECS is in disagreement with the expected deleterious effect of mutations in functional RNA structures.

We developed a Random Forest (RF) model to predict purifying selection at any genome position based on the features present at this position. To this aim we associated every non-coding genome position to a vector of binary values describing the presence/absence of functional features at this location (see S1 Table and Methods). Following feature selection and cross-validation, we obtained a robust model associating any combination of 16 genomic variables to a predicted rare SNP ratio. A measure of importance of each feature's contribution to the RF model is shown in Fig 1B. Evolutionarily conserved regions, promoters and conserved transcription factor binding sites are among the strongest contributors to rare SNP ratio, in line with previous studies [10]. Of note, the predictive value of a high recombination rate, which is associated to a low rare SNP ratio (Fig 1A), had not been reported before.

To evaluate how the SNP model alone can predict deleterious mutation in the non-coding genome, we compared the average scoring of one million random SNPs to that of non-coding variants from two distinct collections of disease-related mutations, the Clivariant [28] and HGMD [29] databases (Fig 1C). Known clinical variants from either database have significantly higher scores by the SNP model than random variants (Wilcoxon P<2.2e-16 in both cases). Furthermore, scores in the SNP model are positively correlated to the density of disease-related SNPs (Fig 1D, r = 0.80 and 0.73, P = 6.09e-08 and 3.15e-06 for Clivariant and HGMD, respectively), which confirms the capacity of the SNP model to identify non-coding regions where mutations are more likely to be disease-related.

### Scoring mutations with the somatic (SOM) model

**Fig 2:**
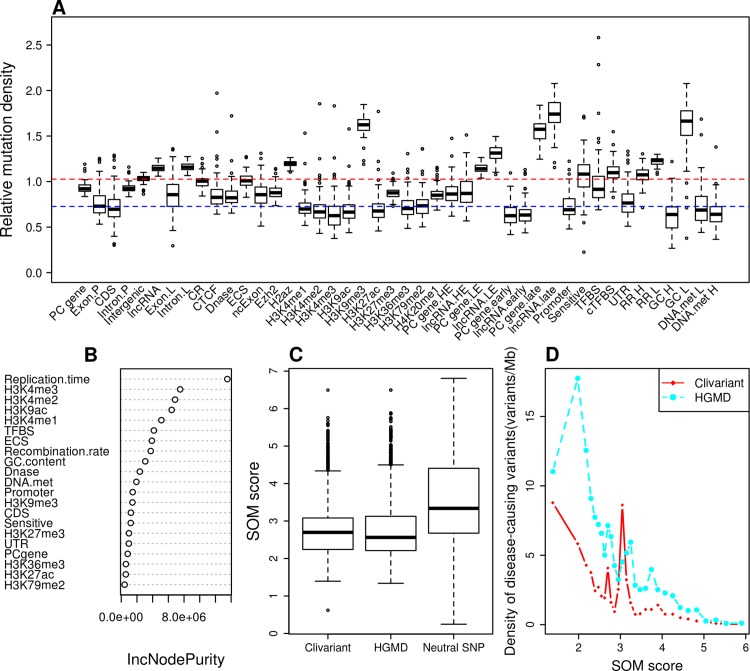
Construction of the Somatic Mutation (SOM) model for liver cancer. **A**. Relative density of somatic mutations from whole genome sequences of 88 liver tumors [11], associated to different genome features (see Methods for feature details). Mutation density is normalized so that the whole genome average has a mutation density of 1. PC gene: protein coding gene; CDS: coding sequence; Exon.P, Intron.P, Exon.L,Intron.L are exon and intron of protein coding gene and lncRNA respectively; CR: conserved region; DNase: DNase I hypersensitive site; ECS: evolutionarily conserved structure; ncExon: non-coding exon; PC gene.HE, LncRNA.HE, PC gene.LE and LncRNA.LE are high expressed and low expressed protein coding gene and lncRNA; PC gene.early, LncRNA.early, PC gene.late and LncRNA.late are early and late replicated protein coding gene and lncRNA; cTFBS: conserved transcription factor binding site;RR H,RR L,GC H,GC L,DNA.met H and DNA.met L are 1-Kb windows with high recombination rate (> 4.0), low recombination rate (< 0.5), high GC content (GC % > 50%), low GC content (GC%<30%), high DNA methylation (average value > 0.7245) and low DNA methylation (average value < 0.4062) respectively; Blue and red dotted lines: base lines showing average values for CDS and intergenic regions, respectively; **B:** Feature importance as measured by IncNodePurity. We only show here features that passed feature selection. **C**. Distribution of SOM scores for neutral SNPs and for clinical variants from two disease-causing variants databases Clivariant and HGMD. Neutral SNPs here are SNPs from the 1000 Genome project with allele frequency higher than 0.01, SOM scores predicted by the random forest model were divided by the number of patients. **D**. Correlation of SOM score with densities of disease-causing variants. Genome positions were sorted by SOM score and split into 100Mb intervals. The plots show the average SOM score and density of disease-causing variants for each interval. The purple dotted line shows cutoff used for defining low SOM score thereafter.

The tumor mutational landscape results from the combined action of multiple factors including mutagenic agents, accelerated cell division, impairment of DNA replication/repair pathways and resistance to treatment [13]. The tumor genome is thus subject to a set of constraints that are quite distinct from those acting in the germline. To analyze these constraints, we collected somatic mutation data from whole genome sequencing of liver cancer (N = 88 patients), chronic lymphocytic leukemia (CLL) (N = 28), lung adenocarcinoma (N = 24) [11] and melanoma (N = 25) [30]. We analyzed mutation densities for the above genomic features and for tissue-specific features such as histone marks, early/late replicated regions and transcript abundance obtained from tissue-matched Encode cell lines [27](S2 Table). Results are shown in Fig 2A, S1A Fig, S2A Fig and S3A Fig. Protein-coding sequences (CDS) harbor relatively low somatic mutation densities compared to introns (intron.P) and intergenic regions in all four cancer types, consistent with higher functional constraints in CDS, as observed in the SNP model. However, other features reveal a quite different pattern. Evolutionary conserved regions, cTFBS and UTRs that were all under strong selective constraints in the germline model present highly variable mutation densities in tumors, with densities ranging from low (CDS level) to high (intergenic level), and no consistent pattern from tumor to tumor (Fig 2A, S1A Fig, S2A Fig and S3A Fig). Certain features, however, present marked and consistent mutational patterns across all four tumors. For instance, we observed an obvious trend for accelerated mutation rates (higher density) in regions of repressed chromatin marks (H3K9me3), late replication (PCgene.late, lncRNA.late), low transcript expression (PCgene.LE, lncRNA.LE) and low GC (GC L). Conversely, we observed consistently reduced mutation rates in regions of active chromatin marks (H3K4me1-2-3, H3K79me2, H4K20me1), early replication (PCgene.early, lncRNA.early), high transcript expression (PCgene.HE, lncRNA.HE) and high GC (GC H). The general trends in feature-wise mutation densities largely reflect prior findings based on smaller datasets. Schuster-Bockler and Lehner [25] observed strong correlations between chromatin states and mutation densities in tumors, with repressive marks linked to higher mutation rates, possibly due to deficient DNA repair in these regions. Mutation density is also known to correlate positively with late replication [13,31,32] and negatively with recombination rate [25] and RNA expression level [33][13].

To model the mutational constraints acting on the tumor genome, we developed a second RF model, referred to as the SOM model, which predicts somatic mutation densities (the response variable) at any genome position based on the presence of cell-specific and generic genome features. We built one SOM model for each of the four above cancer types. Due to the large number of features in the SOM model and limited number of somatic mutations in the training sets, we computed feature coverage or average values (see methods) on successive 1Mb regions and trained the RF model based on the resulting vectors. After feature selection and robustness testing by cross-validation, the SOM model enabled reliable prediction of somatic mutation density at any genome location for each cancer type (see Methods). Fig 2B, S1B Fig, S2B Fig and S3B Fig show the importance of features in the SOM models.

RNA expression levels turned out to be relatively weak predictors of mutation density, whereas replication time and histone marks in general were the predominant features determining somatic mutation density in all cancer types. However we observed significant differences between cancers. For instance the H3K36me3 mark is an important predictor of low mutation density in melanoma and lung cancer, not in CLL or liver cancer. Also, CTCF binding sites are strong predictors of low mutation density in CLL and not in other cancer. Altogether this indicates that each somatic model predicts a cancer-specific mutation profile with distinct regions of high and low mutation densities.

Under a neutral evolutionary model, somatic mutations should freely accumulate in regions that do not impact tumor fitness, thus regions of elevated tumor densities (high SOM score) should be considered as generally irrelevant to fitness, while regions that are relatively preserved from somatic mutations (low SOM score) are potentially the most interesting as they could reveal purifying selection occurring at the tumor level. One way to test this hypothesis is to relate low mutation regions and the occurrence of known disease mutations. Fig 2C, S1C Fig, S2C Fig and S3C Fig show that non-coding disease mutations from the Clivariant and HGMD databases have significantly lower SOM scores than evolutionarily neutral SNPs (Wilcoxon P<2.2e-16 in all cases). Furthermore, the SOM score of different genome regions is inversely correlated to the density of disease causing variants in these regions (Fig 2D, S1D Fig, S2D Fig and S3D Fig) (r = -0.47 to -0.94, P = 0.01 to 8.61e-14) suggesting that genome regions spared from somatic mutations are functionally relevant to disease progression.

To further assess the value of SOM score as an indicator of selection, we mapped the genome positions with lowest SOM scores onto the different genome features and measured the relative enrichment for low SOM score positions within each feature (S4A Fig). Expectedly, features that were part of the SOM model are significantly enriched or depleted in low SOM scores. However, 5' and 3' splice sites, two features that were not part of the model, show a much higher coverage by low SOM score regions than intronic regions, which indicates functional non-coding elements tend to attract fewer somatic mutations, as expected under a negative selection model. This effect is also observed in lncRNA, consistent with the higher conservation of splice junctions in this class of genes [34]. Conversely, features enriched in high SOM scores (S4B Fig) predominantly correspond to silent regions (intergenic, centromeres and telomeres). In summary low SOM score positions tend to colocalize with functional elements and correlate with disease-causing mutations, suggesting the SOM model could be a significant, independent source of functional information on non-coding regions.

### Towards an integrated model for germline and somatic mutations

Analysis of germline and somatic mutations suggests that each mutational regime carries valuable independent information about selective forces acting in a tumor. We thus questioned whether combining SNP and SOM information at each genome position may lead to improved mutation prioritization in cancer.

**Fig 3:**
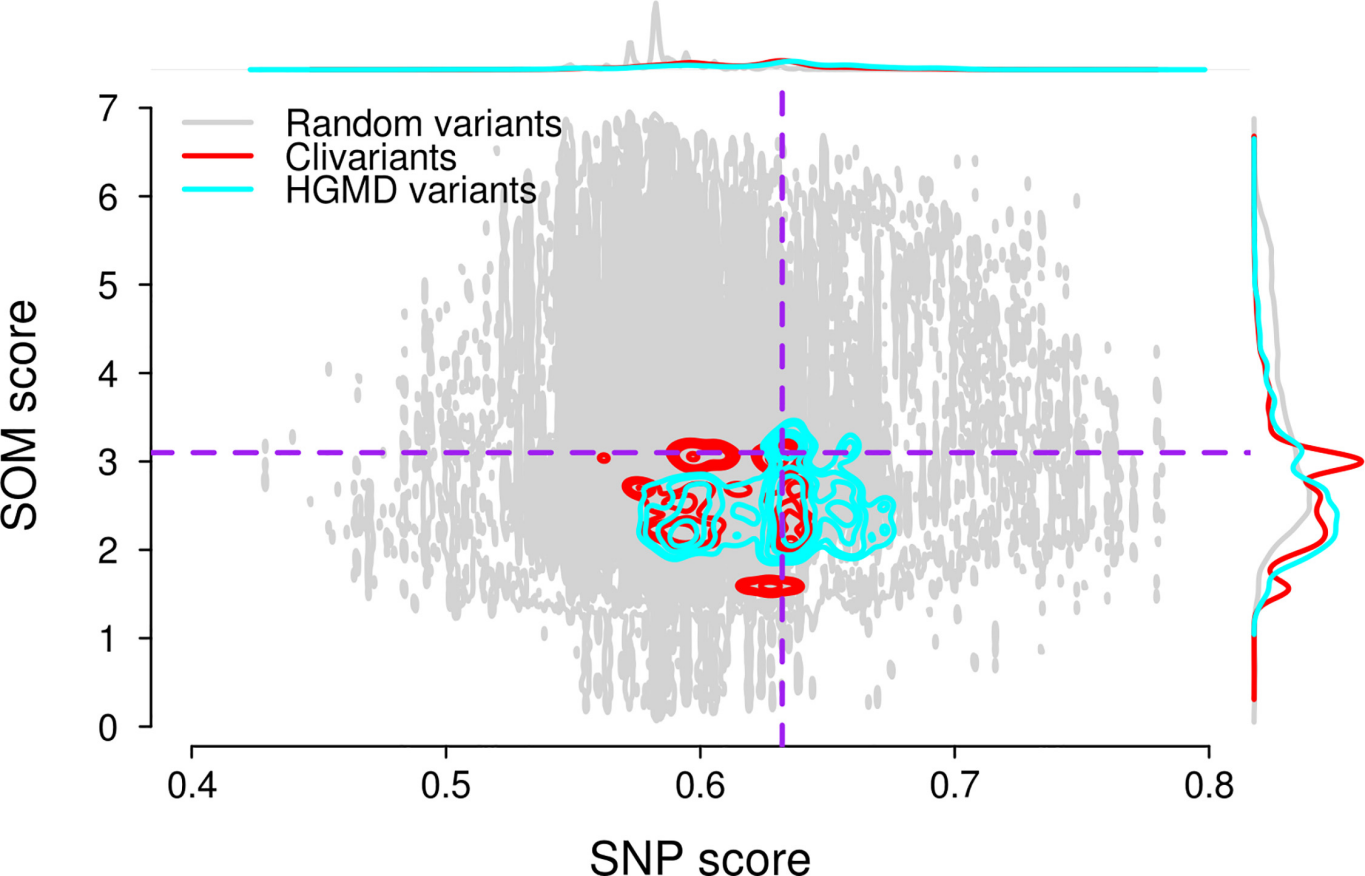
Relationship between SNP and SOM scores in liver cancer. Grey dots: 1 million random genome positions; cyan contour: HGMD disease-causing variant positions; red contour: Clivariant positions. The top and right curves show marginal distributions of SNP scores (top) and SOM scores (right) for random genome positions, HGMD and Clivariant disease-causing variant positions. Dotted lines define cutoff values for hypomutated/hypermutated regions. SNP score cutoff = 0.63 (98.16Mb above cutoff), SOM score cutoffs = 3.10 variants/Mb, defining areas below cutoff of 55.67 Mb, in liver cancer. Hypomutated regions defined by both cutoff correspond to ~56Mb in liver cancer type.

Fig 3 presents the relationship between SNP and SOM scores for one million random genome positions and for known clinical variants. Although SNP and SOM scores are generally uncorrelated, the roughly triangular shape of the right side of the spectrum (pointing to the lower-right) shows that strong purifying selection (high SNP score) tends to associate with low tumor mutation rates (low SOM score). Disease-causing variants are loosely concentrated in this lower-right corner, consistent with these positions being both under negative selection in the germline and relatively preserved from mutational damage in the tumor. This region can be empirically delineated by a SNP score cutoff encompassing the 100Mb highest scores (constant for all cancer types) and a SOM score cutoff defined in such a way that most disease causing mutations are found below this cutoff in liver cancer (Fig 3 dashed line). These criteria define two regions of interest in the high SNP score area of the mutational spectrum, that we will term "hypomutated" and "hypermutated", with reference to somatic mutations (Fig 3). We set the SOM cutoffs in the different cancer types (S5 Fig) so that the hypomutated area has the same size (56Mb) as in liver cancer.

To assess the benefits of the joint model for scoring disease mutations, we measured disease variant densities in different areas of each tumor spectrum using the above cutoffs (S3 Table, S6 Fig). If we intersect high-SNP and low-SOM regions, the resulting genome area shows a greater enrichment in disease variants than either region taken independently (χ^2^ P<2.2e-16 for all four cancers). Therefore we argue that integrating germline and somatic mutational models provide a better system for prioritizing damaging mutation than any model used independently.

**Fig 4:**
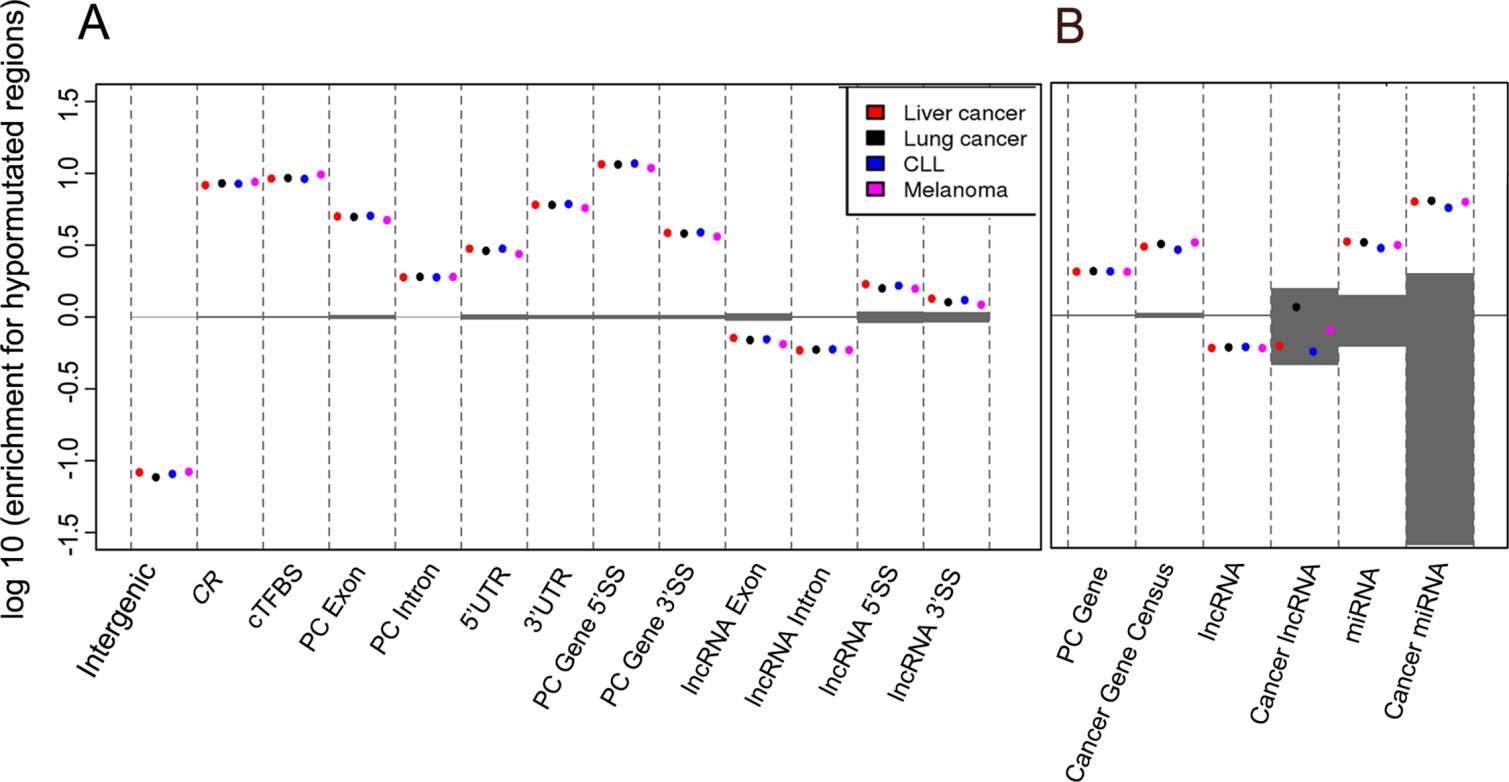
Enrichment for hypomutated positions within different genome features (A) and gene classes (B). Positive values indicate enrichment, negative values indicate depletion. Hypomutated (high SNP, low SOM) positions were mapped onto genome features (A) or genes from three different classes (Protein-coding, lncRNA, miRNA) (B). For each feature or gene class, enrichment for hypomutated positions was computed as explained in Methods. As hypomutated positions are cancer-specific, different results are obtained for each cancer class (colored dots). Shaded grey areas show enrichment ranges obtained from 1000 random permutations (see Methods).

Hypomutated positions are significantly over-represented within splice junctions, UTRs and different classes of cancer genes. We mapped predicted hypomutated positions on different genome features and gene types (Fig 4). As expected, functional features of protein-coding genes such as intron junctions and UTRs are strongly enriched for hypomutated positions (Fig 4A). Similar trends are observed in lncRNA genes. Both lncRNA introns and exons are generally depleted for hypomutated regions (Fig 4), in line with poor selective pressure in lncRNA overall. However, lncRNA splice sites are slightly, albeit significantly, enriched in hypomutated regions, consistent with previous studies showing increased purifying selection at lncRNA splice sites [34].

We then compared hypomutated position enrichment in cancer vs. non-cancer genes. Cancer-related protein-coding genes and cancer-related miRNAs are enriched for hypomutated regions compared to their non-cancer counterparts (Fig 4B, S4 Table). This result suggests an elevated protection from somatic and germline mutations in cancer miRNAs and in the introns and UTRs of known cancer genes (we remind our analysis only considers the non-coding part of genes). However, we did not observe a significant enrichment for hypomutated regions in our short list of cancer-related lncRNAs (N = 25). Complete lists of protein-coding, lncRNA and miRNA genes with their fraction of hypomutated positions are provided as S1 Dataset. Notable cancer genes with high fractions of hypomutated positions include PIM1 and MED12, with respectively 34% and 32% of their non-coding length that is hypomutated. Among cancer miRNAs, miR-1 and miR-574 are both covered almost completely by hypomutated positions.

Interestingly, genes with high fractions of hypermutated positions are more divergent between cancer types than genes with high fractions of hypomutated positions (S7 Fig), suggesting areas of high mutation density are largely cancer-specific, while areas of low mutation density tend to locate in the same functional regions of the genome. GO-term biases in these gene sets are significant only for genes enriched for hypermutated positions in liver cancer and CLL, and involve transcription regulation functions (S5 Table).

## Discussion

We introduced novel computational models to assess mutational constraints in the non-coding genome based on the presence of functional features. We trained a model on germline SNP data to predict rare SNP ratio at any genome site, and we trained four cancer-specific models on tumor data to predict somatic mutation densities. These models thus provide two independent measures of mutational constraints that are both relevant to the analysis of non-coding regions in the cancer context. Furthermore, the feature-based model construction enabled us to analyze the contribution of each feature to the germline and tumor mutation landscape and to characterize the main differences between the two mutational regimens.

A major point we want to highlight in this study is that combining germline and somatic data provide an improved definition of non-coding regions that are sensitive to mutation in cancer cells. To illustrate this point, we extracted genome areas combining a high rare SNP ratio and a low somatic mutation density and showed these combined criteria are a better predictor of disease causing mutation than rare SNP ratio or somatic mutation density considered independently.

Distinctly from current models that consider somatic mutation only as a corrective mean to avoid overpredicting deleterious mutations in highly mutated regions [10,13,22], our approach thus considers somatic mutations on a par with evolutionary mutations, that is as a criterion to tell apart genome positions that are neutral (highly mutated) or under purifying selection (lowly mutated) in the tumor genome. We remind that prevalent forces shaping the tumor mutation landscape are the combined actions of mutagens and the DNA repair machinery on differentially accessible genome regions [25,35,36]. Therefore, if functional areas are relatively spared from mutation, this is mostly not as a result of purifying selection, but because they are under the closer watch of DNA repair systems. Hence the somatic model can be viewed primarily as a way to discard regions sustaining accelerated mutations. However, we showed that hypomutated regions were enriched in functional elements such as splice junctions, which suggests purifying selection may occur as well.

We are aware of the limited accuracy of somatic models when these are trained over tumors with low mutation rates and/or few available whole genome datasets. Currently, there are far fewer mutations to learn from in the tumor dataset than in the human polymorphism dataset (aggregate mutation densities in the present cancer datasets ranged from 20 to 600 mutations per Mb, v*s*. >12,000 SNP per Mb in the 1000 Genome data). This limits our ability to observe small-scale variations in mutation density. We expect that the fast accumulation of whole tumor sequences will improve model accuracy within each cancer type and provide independent validation of our approach on other tumor classes. Another potential limitation in SOM models is the use of expression and epigenetic features from cell lines as a proxy for cancer tissues. This should also improve in the future as such information is acquired from primary tumor tissues.

A key outcome of our study is a new approach to prioritize non-coding variations for cancer driver search. Our models predict mutational constraints at a genome position based on generic features, that is, largely independently of the actual mutations observed at this specific location. Therefore, a locus may be predicted as hypomutated by the model and yet turn out to sustain recurrent mutations across patients. Such a locus should then be prioritized as a candidate driver. Such analyses will be natural extensions of the present study.

Although cancer research now acknowledges the importance of non-coding drivers, the search for cancer-related mutations has focused on regulatory elements such as promoters and enhancers as the key non-coding elements [10,24]. The realization that nearly 60,000 lncRNAs are expressed, often specifically, in tumoral genomes, many of them harboring potential disease causing mutations [4], combined to the regulatory roles played by many lncRNAs [37] indicate that cancer driver search should also encompass those larger transcribed regions. Even if only 10% of lncRNAs are functional by conservative estimates [5], this corresponds to a much larger genome area than known regulatory elements. Currently, the search for cancer genes in these non-coding RNAs is driven by expression signature analysis. We show here that the analysis of germline and somatic mutational regimen is an important alternative that may lead to the identification of cancer-driving elements in ncRNA genes, as well as in the non-coding fraction of mRNA genes.

## Materials and Methods

### Human polymorphism, mutation and disease data

Human polymorphism data comprising 38,248,779 SNPs were downloaded from the 1000 Genome project pilot 1 [21] (http://www.1000genomes.org). The data set contains SNP data from 2500 individuals from about 25 world populations. SNPs with allele frequency lower than 0.01 were defined as rare, other SNPs were considered neutral.

Somatic variants were collected from whole genome sequencing of paired cancer and normal tissues, obtained from two studies: 2,011,261 variants from 25 melanoma patients [30], 1,845,976 from 24 lung adenocarcinoma patients, 881,136 from 88 liver cancer patients and 59,993 from 28 chronic lymphocytic leukemia (CLL) patients [13]. Variants described as "substitution" or "indel" were both collected and are referred to collectively as mutations in the text.

Curated disease-related variants were obtained from the Clivariant (Version 2014/03/03, 55,689 variants) [28] and HGMD (Version 2014/04/14, 166,768 variants) databases [29]. After exclusion of coding positions we used 13,108 HGMD and 6045 Clivariant mutations.

Lists of cancer genes for Fig 4 were obtained as follows: protein-coding cancer genes are from the Cancer Gene census, available from COSMIC release V71 (http://cancer.sanger.ac.uk/cancergenome/projects/census/) [38]; cancer-related lncRNAs are 25 mammalian long non-coding transcripts identified from our literature search as experimentally associated with different cancer types (S6 Table); cancer miRNAs are from the miRCancer database [39].

### Uniform genome-wide features

Uniform features used in all figures and models are summarized in S1 Table. Human genome annotation (protein-coding and lncRNA genes, exons, introns, CDS, UTRs, non-coding Exons (ncExon) was obtained from Gencode V7 [3]. We defined as intergenic those regions covered by neither a protein-coding gene (including introns) nor an lncRNA. We defined as 5’ and 3' splice sites intron regions spanning the first 10 nt on the 5' side and the last 50 nt on the 3' side. GC contents were computed directly from the HG19 human genome assembly. We defined 1kb regions with > 50% GC as high GC and 1kb regions with < 30% GC as low GC. For the SOM model, GC contents were computed over 1Mb windows.

Promoters, defined as regions of 2.5kb from transcription start site (TSS), are from the Gerstein lab (http://funseq.gersteinlab.org/data) [10]. Enhancers are from the Atlas of active *in vivo*-transcribed enhancers, collected based on FANTOM5 CAGE data from multiple tissues and cell lines [40]. TFBSs combine all transcription factor binding sites from more than 30 Encode cell lines [27]. Conserved TFBS (cTFBS) are from the UCSC tfbsConsSite track established from human/mouse/rat alignment [41].

"Sensitive regions" are defined in the Khurana et al. study of genome regions under purifying selection as the 0.4% genome fraction with highest enrichment in rare SNPs [10]. Evolutionarily conserved regions (CR) are from the UCSC 46 mammalian genome alignment (Phastcons score >177) [41]. Evolutionarily conserved structures (ECS) are RNA secondary structures predicted using comparative structure prediction algorithms based on multiple genome alignments [42]. DNase I hypersensitive sites (DNase I) from 125 combined ENCODE cell lines were obtained directly from the UCSC web site [27].

We defined early and late replication regions using the ENCODE ‘Repli-seq' track (http://genome.ucsc.edu/ENCODE) that provides signals for cell cycle fractions G1b, S1, S4, G2 in different cell types [27]. For each protein-coding or lncRNA gene, we computed the early-to-late (E/L) ratio as (G1b+S1)/(S4+G2) averaged over the gene length. Early and late replicated genes denote genes or lncRNAs with an E/L ratio > 1 or < 1 for all 10 cell lines respectively: Gm12878, Hela3, Hepg2, Mcf7, Imr90, K562, Bg02es, Huvec, Bj and SK-N-SH.

Expression levels were calculated using number of reads per kilobase per million reads (RPKM). We defined as High Expression (HE) genes those with RPKM > 20 in any of the 27 Encode cell lines [27], corresponding to the top 6% of protein coding genes for a single Encode cell line.

Recombination rates (RR) are from the International HapMap Project (http://hapmap.ncbi.nlm.nih.gov/) [43]. As every genome position did not have an associated RR, we averaged HapMap RR values over 1kb windows. High replication rate (RRH) and low replication rate (RRL) regions were defined by an average replication rate above 4.0 or below 0.5, respectively.

### Tissue-specific features

RNA expression levels, transcription factor binding sites (TFBS) and maps of histone modification marks were acquired from UCSC ENCODE tracks [27] for each cell type: Hepg2, A549, K562, Nhdfad (S2 Table). Replication timings were acquired from UCSC ENCODE tracks for cell lines Hepg2, lmr90, K562, Bg02 (S2 Table).

To define high expression and low expression genes, expression levels were measured for a single randomly selected cell line from the same tissue for each independent protein coding gene and lncRNA. RPKM values above 20 and below 0.25 defined high (PCgene.HE, lncRNA.HE) and low expression genes (PCgene.LE, lncRNA.LE), respectively.

Replication timings were defined for each protein-coding gene and lncRNA using the same E/L calculation as above. Genes with an E/L ratio > 1 were considered early replicated (lncRNA.early, PCgene.early), genes with an E/L ratio < 1 were considered late replicated (lncRNA.late, PCgene.late).

DNA methylation data were obtained from TCGA database for cancer types liver hepatocelluar carcinoma, lung adenocarcinoma, acute myeloid leukemia and skin cutaneous melanoma. Average DNA methylation value was computed for each methylation site across multiple patients using available values. Undefined values were then replaced with mean and we averaged DNA methylation over non-overlapping 1Kb and 1Mb windows. 1Kb windows with mean DNA methylation values greater than 0.7245 and less than 0.4062 were defined as high (DNA.met H) and low (DNA.met L) DNA methylation windows respectively.

### Rare SNP model

A random forest (RF) is an ensemble of multiple decision trees computed from separate bootstrap samples of the training data and feature set [44]. We developed the germline RF model (SNP model) to predict the density of rare SNP at any genome location based on 14 distinct features (S1 Table). The response variable was the local ratio of rare SNP (number of rare SNPs /total number of SNPs) obtained from the 1000 Genome Project.

A matrix of 44130 rows was formed after removal of those combinations in coding regions, each row representing one type of combination of features that can be observed throughout the non-coding genome. Feature selection was performed with the R VSURF package [45], resulting in elimination of features GC (S1 Table) and late replicated regions, leaving 18656 combinations of the remaining 16 features. 2502 combinations of 16 features containing 99.49% of SNPs and 99.50% of the human genome were used to train the model after removal of the combinations of size smaller than 10Kb. The RF model was produced using the R randomForest package. The SNP score was predicted with the 16 selected features for each combination of feature in the non-coding genome. Model calibration and cross validation are presented in S1 Text. Variable importance (Fig 1B) was estimated using node purity, which measures the decrease in tree node purity that results from splits of a given variable.

### Somatic mutation model

The somatic (SOM) RF model was built using as predictors the 16 uniform and 17 tissue-specific features described in S1 Table and S2 Table, and as response variable the local density of somatic mutation across all tumors in the cancer type under study. Due to the relatively sparse somatic mutation data, model fitting was performed using continuous variables measured for genome windows as explained below.

Features ncExon, introns of lncRNAs and PC genes, CR, cTFBS, UTR, Promoter, GC contents and the various histone marks were expressed as the number of nucleotides covered by the feature within each successive 1Mb window. Features recombination rate, DNA methylation, replication time and expression level were computed for each successive 1Mb window as follows. To obtain expression levels for 1Mb windows, RNA-seq reads from each cell lines (3 samples/cell line) were counted, and the length of exons from Gencode annotation was calculated, then, average expression level was calculated as RPKM. Replication time in the SOM model was the average E/L ratio computed as above for each 1Mb window. Recombination rate and DNA methylation were averaged over non-overlapping 1-Mb windows across the genome.

The SOM model used cancer mutation density as the response variable and the 33 genomic features (32 for lung cancer) as predictor variables. A matrix of 2846 rows was formed, of which each row represents a 1-Mb window and columns contain values of genomic features and response variable. For model fitting, we discarded genome regions with poor annotation or biased mutation information. This included any 1Mb window overlapping a telomere, centromere, stalk, pericentromere, or with 100% undefined bases, and the entire Y chromosome due to ploidy bias (total: 224.3Mb). All predictor values were plus one and log scaled.

The RF regression model was constructed with the R randomForest package as above. Feature selection was performed with the R VSURF package [45]. Model calibration, robustness testing/cross validation of the SOM models are presented in S1 Text.

For SOM score prediction, we used the same 1-Mb window strategy as in model building, however, the 1Mb-windows were slided across the human genome with a step size of 1Kb, in order to extrapolate to regions not used in model building. 1Mb windows unfit for model training were excluded as above, resulting in 2,832,687 overlapping 1Mb window annotations. The SOM score was predicted using selected features for each 1Mb window and averaged on a 1 Kb window scale.

### Enrichment analysis

Enrichment for hypomutated positions within different feature classes (Fig 4) was measured as the odds ratio:

$$enrichment=\frac{\left(\frac{Hf}{Sf}\right)}{\left(\frac{Hg}{Sg}\right)}$$

Where *Hf* = #hypomutated positions within feature, *Sf* = total size of feature, *Hg* = #hypomutated positions in whole genome, *Sg* = total size of genome. The significance of enrichment or depletion was evaluated using a permutation test as follows: a set of positions of same size as the hypomutated region (ie. 56Mb) was randomly sampled from the whole genome 1000 times, and in each random sample, enrichments were calculated for each feature class. The distribution of enrichment values from the 1000 random samples is shown as shaded areas in Figures. Only observed enrichments outside these areas are considered significant. Enrichment for other types of positions (hypermutated, low SOM score etc.) was evaluated similarly.

## Supporting Information

S1 Fig Construction of the Somatic Mutation (SOM) model for Lung adenocarcinoma. (DOCX)

S2 Fig Construction of the Somatic Mutation (SOM) model for CLL. (DOCX)

S3 Fig Construction of the Somatic Mutation (SOM) model for melanoma. (DOCX)

S4 Fig Enrichment for low SOM score or high SOM score positions within genome features in the four cancer types. (DOCX)

S5 Fig Effect of combining high SNP scores and low SOM scores in 4 cancer types. (DOCX)

S6 Fig Venn diagrams showing the distribution of genes covered by hypomutated or hypermutated positions across the 4 cancer types. (DOCX)

S7 Fig Venn diagrams showing the distribution of genes covered by hypomutated (A) or hypermutated (B) positions, across the 4 cancer types. In each cancer type the 100 genes with the highest coverage by hyper/hypomutated regions is shown.
(DOCX)

S1 Table Uniform genomic features used in figures and SNP or SOM models. (DOCX)

S2 Table Cell-specific genomic features used in figures and SOM models. (DOCX)

S3 Table Significance of disease mutation enrichment in high-SNP+low SOM regions. (DOCX)

S4 Table Significance of over-enrichment for hypomutated regions within cancer vs non-cancer genes. (DOCX)

S5 Table GO-term biases in protein coding genes selected for the presence of hypomutated or hypermutated elements. (DOCX)

S6 Table Compilation of cancer long non-coding RNAs. (DOCX)

S1 Dataset Tables of protein-coding, small RNA and lncRNA genes overlapping hypomutated regions for each cancer type. **The total size and fraction of overlap (gene size/overlap size) were computed for each gene. Columns 1–7 correspond to chromosome, gene start, gene end, gene name, size of overlap and fraction of overlap. Genes with no overlap are excluded. All coordinates use HG19 genome assembly.** pcgenemap-liver.tsv: protein coding genes, liver cancer; pcgenemap-lung.tsv: protein coding genes, lung cancer; pcgenemap-CLL.tsv: protein coding genes, CLL; pcgenemap-melanoma.tsv: protein coding genes, melanoma; lncRNAmap-liver.tsv: lncRNA genes, liver cancer; lncRNAmap-lung.tsv: lncRNA genes, lung cancer; lncRNAmap-CLL.tsv: lncRNA genes, CLL; lncRNAmap-melanoma.tsv: lncRNA genes, melanoma; sRNAmap-liver.tsv: small RNA genes, liver cancer; sRNAmap-lung.tsv: small RNA genes, lung cancer; sRNAmap-CLL.tsv: small RNA genes, CLL; sRNAmap-melanoma.tsv: small RNA genes, melanoma. *R scripts*: RFmodel_workflow.R; RF_SOM_validation.R; RF_SNP_validation.R.
(ZIP)

S1 Text Random Forest models; Model Calibration; Feature Selection; Model Validation. (DOCX)
