# Supplementary material for: A Dual Model for Prioritizing Cancer Mutations in the Non-coding Genome Based on Germline and Somatic Events
Source: PLoS Comput Biol. 2015 Nov 20;11(11):e1004583. doi: 10.1371/journal.pcbi.1004583 (PMC4654583; doi:10.1371/journal.pcbi.1004583)
Supplement: S1 Fig — (DOCX) [file pcbi.1004583.s001.docx]

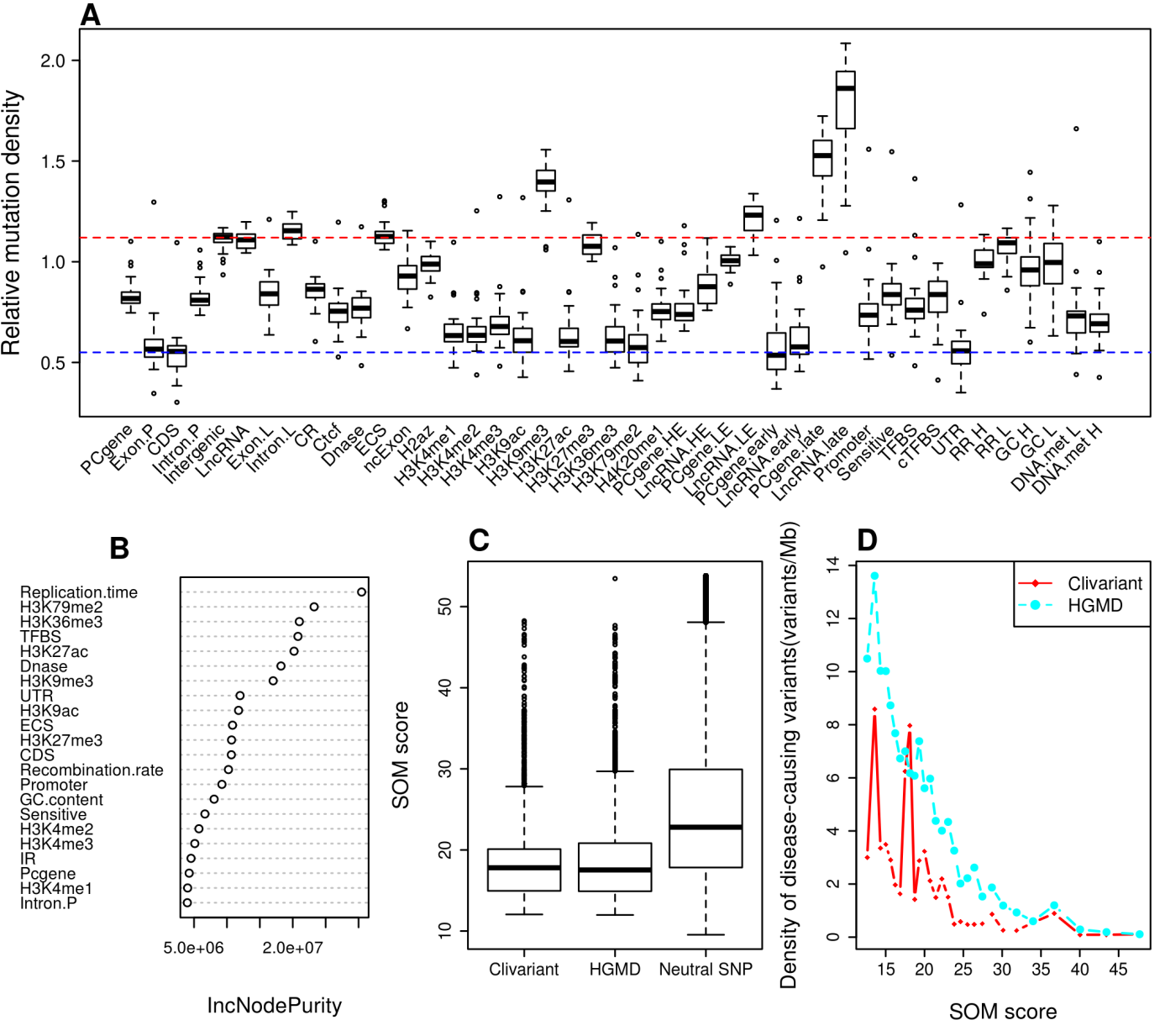
Figure S1. Construction of the Somatic Mutation (SOM) model for lung cancer. **A**. Relative density of somatic mutations from whole genome sequences of 24 lung cancer, associated to different genome features (see Methods for feature details). Mutation density is normalized so that the whole genome average has a mutation density of 1. PCgene: protein coding gene; CDS: coding sequence; Exon.P , Intron.P, Exon.L,Intron.L are exon and intron of protein coding gene and lncRNA respectively; CR: conserved region; DNase: DNase I hypersensitive site; ECS: evolutionarily conserved structure; ncExon: non-coding exon; PCgene.HE, LncRNA.HE, PCgene.LE and LncRNA.LE are high expressed and low expressed protein coding gene and lncRNA; PCgene.early, LncRNA.early, PCgene.late and LncRNA.late are early and late replicated protein coding gene and lncRNA; cTFBS: conserved transcription factor binding site; RR H,RR L,GC H,GC L,DNA.met H and DNA.met L are 1-Kb windows with high recombination rate (> 4.0), low recombination rate (< 0.5), high GC content (GC % > 50%), low GC content (GC%<30%), high DNA methylation (average value > 0.7245) and low DNA methylation (average value < 0.4062) respectively; Red and blue dotted lines: base lines from CDS and intergenic regions; **B:** Feature importance as measured by IncNodePurity. We only show here features that passed feature selection. **C**. Distribution of SOM scores for neutral SNPs and for clinical variants from two disease-causing variants databases Clivariant and HGMD. Neutral SNPs here are the SNPs with allele frequency higher than 0.01 from the 1000 Genome project, SOM scores were predicted by the random forest model and divided by the number of patients. **D**. Correlation of SOM score with densities of disease-causing variants. The purple dotted line shows cutoff used for defining low SOM score in lung cancer.
