# Supplementary material for: A Dual Model for Prioritizing Cancer Mutations in the Non-coding Genome Based on Germline and Somatic Events
Source: PLoS Comput Biol. 2015 Nov 20;11(11):e1004583. doi: 10.1371/journal.pcbi.1004583 (PMC4654583; doi:10.1371/journal.pcbi.1004583)
Supplement: S4 Fig — (DOCX) [file pcbi.1004583.s004.docx]

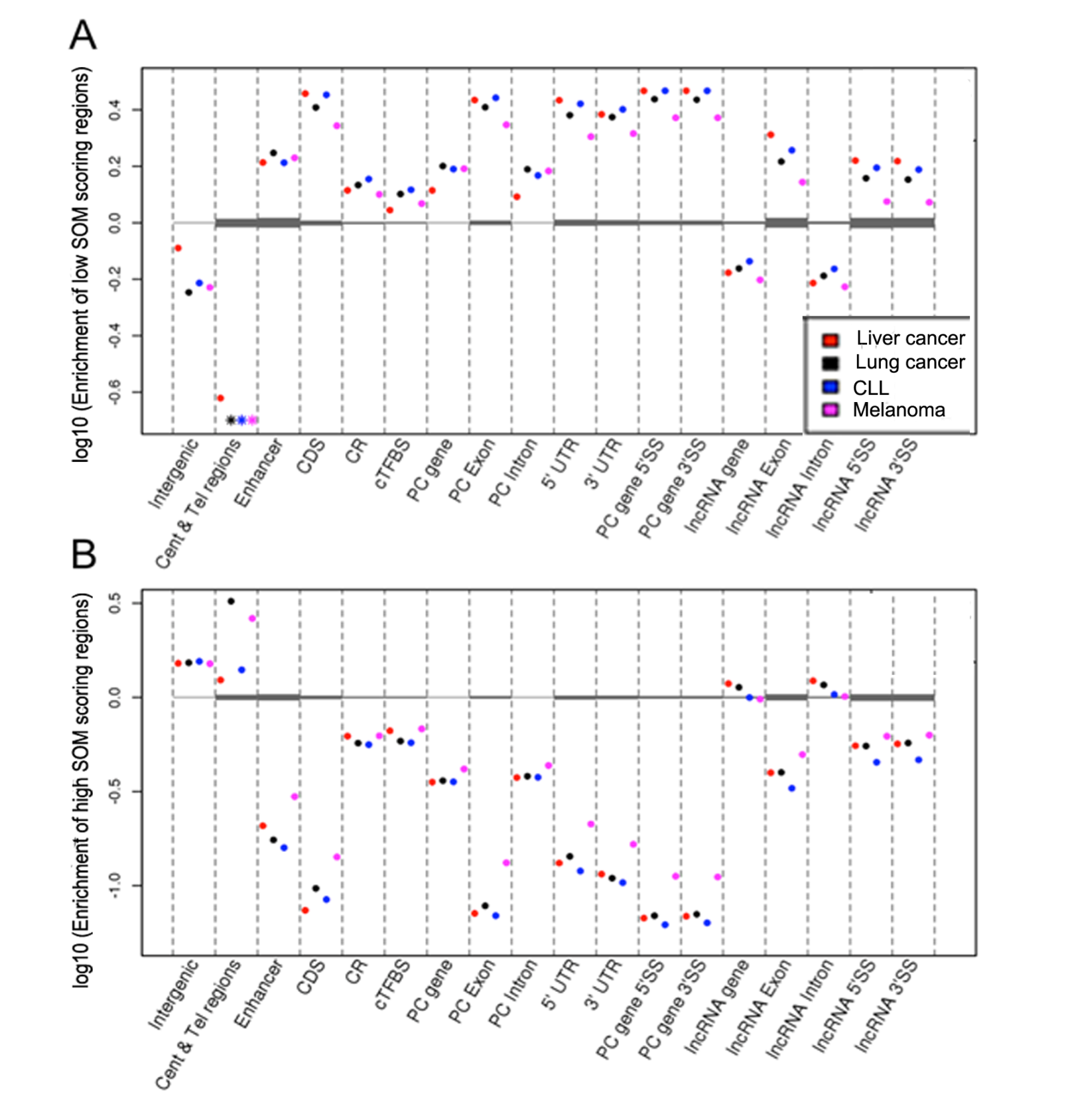


Figure S4. Enrichment for low SOM score (A) or high SOM score (B) positions within genome features in the four cancer types. Low (high) SOM score regions are defined as the 300M positions of the genome with lowest (highest) SOM score. For each feature, enrichment is computed as an odds ratio as explained in Methods. Shaded grey areas show enrichment ranges obtained from 1000 random permutations of the 300M positions (see Methods). Values for each cancer are represented by a dot of distinct color.
