# Supplementary material for: A Dual Model for Prioritizing Cancer Mutations in the Non-coding Genome Based on Germline and Somatic Events
Source: PLoS Comput Biol. 2015 Nov 20;11(11):e1004583. doi: 10.1371/journal.pcbi.1004583 (PMC4654583; doi:10.1371/journal.pcbi.1004583)
Supplement: S5 Fig — (DOCX) [file pcbi.1004583.s005.docx]

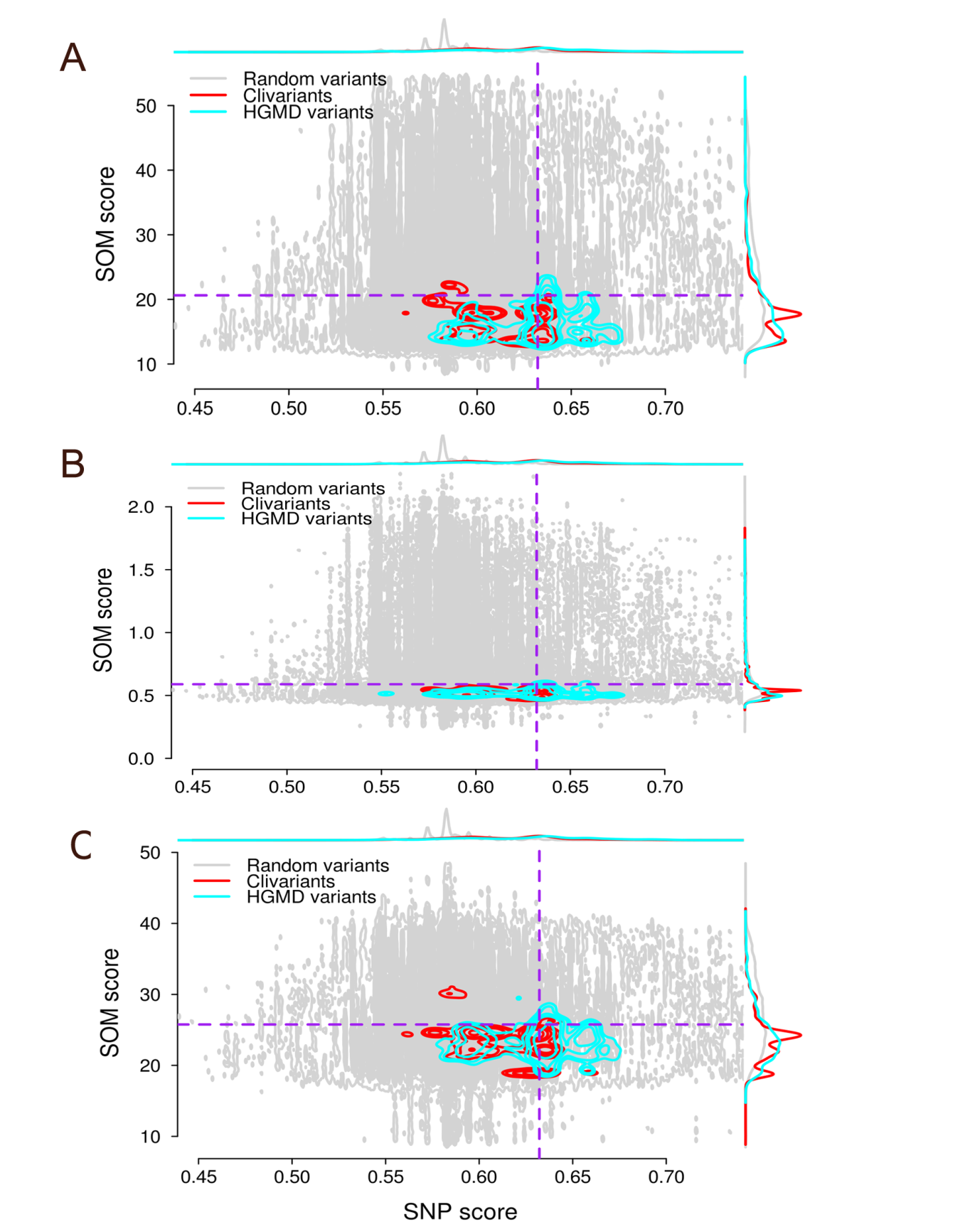


Figure S5. Relationship between SNP and SOM scores in lung cancer (A), CLL (B) and melanoma (C). Grey dots: 1 million random genome positions; cyan contour: HGMD disease-causing variant positions; red contour: Clivariant positions. The top and right curves show marginal distributions of SNP scores (top) and SOM scores (right) for random genome positions, HGMD and Clivariant disease-causing variants. SNP score cutoff=0.63 (100Mb above cutoff), SOM score cutoffs = 20.63, 0.59 and 25.76 variants/Mb, defining areas below cutoff of 1186.45 Mb, 1236.51Mb and 1170.98Mb in lung cancer, CLL and melanoma, respectively. Hypomutated regions (bottom, right area) defined by both cutoffs correspond to ~56Mb in each cancer type.
