# Supplementary material for: A Dual Model for Prioritizing Cancer Mutations in the Non-coding Genome Based on Germline and Somatic Events
Source: PLoS Comput Biol. 2015 Nov 20;11(11):e1004583. doi: 10.1371/journal.pcbi.1004583 (PMC4654583; doi:10.1371/journal.pcbi.1004583)
Supplement: S6 Fig — (DOCX) [file pcbi.1004583.s006.docx]

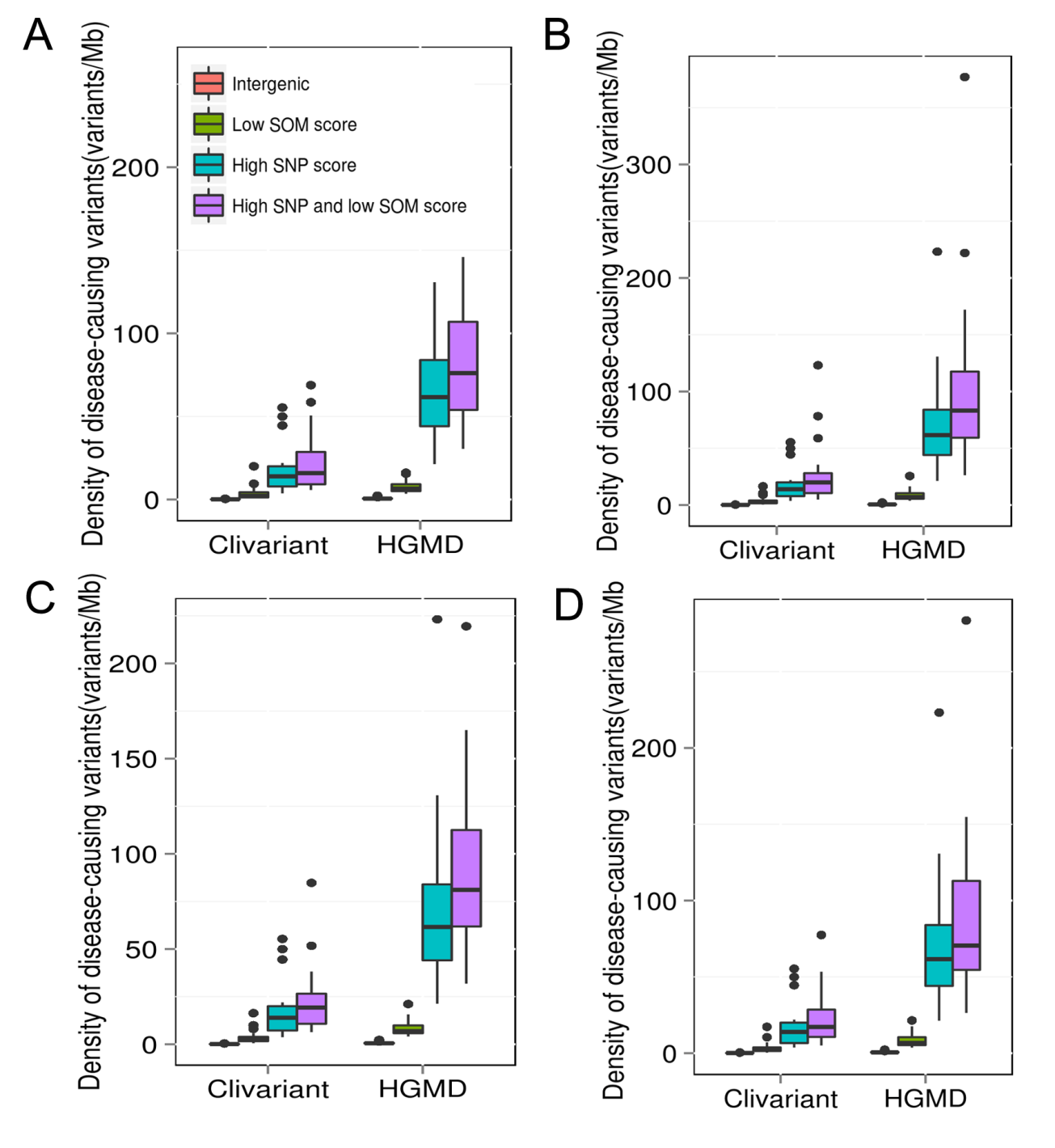


Figure S6. Effect of combining high SNP scores and low SOM scores in 4 cancer types (A: liver cancer, B: lung cancer, C: CLL, D: melanoma). For each chromosome, the size of intergenic, high SNP, low SOM and high SNP + low SOM regions, was calculated and numbers of disease-associated variants either from HGMD or Clivariant were counted. The boxplot shows densities of disease-associated variants in each type of region, chromosome by chromosome. Cutoffs for defining high SNP and low SOM are the same as in Fig 3.
