# Supplementary material for: A Dual Model for Prioritizing Cancer Mutations in the Non-coding Genome Based on Germline and Somatic Events
Source: PLoS Comput Biol. 2015 Nov 20;11(11):e1004583. doi: 10.1371/journal.pcbi.1004583 (PMC4654583; doi:10.1371/journal.pcbi.1004583)
Supplement: S7 Fig — In each cancer type the 100 genes with the highest coverage by hyper/hypomutated regions is shown. (DOCX) [file pcbi.1004583.s007.docx]

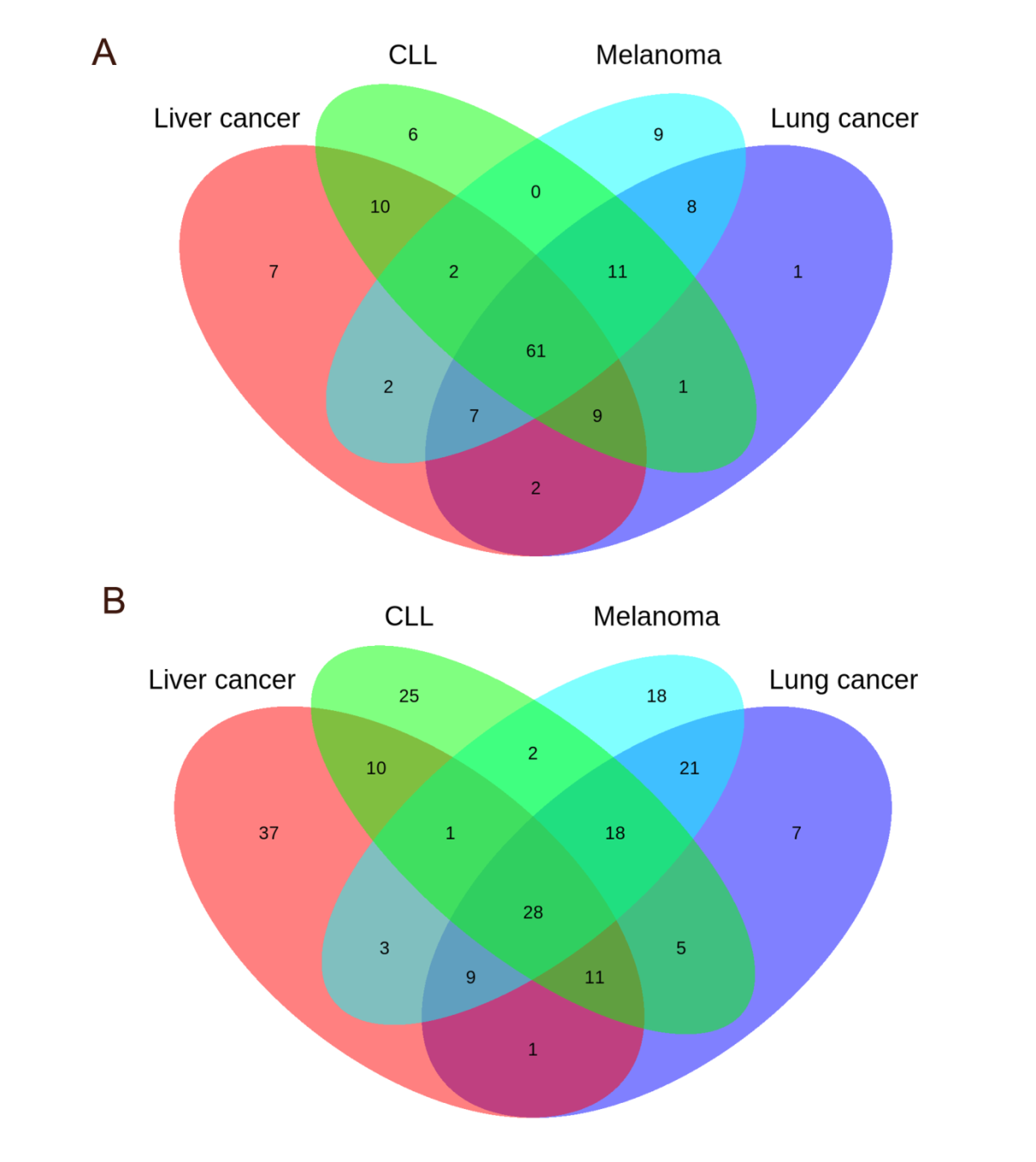


Figure S7. Venn diagrams showing the distribution of genes covered by hypomutated (A) or hypermutated (B) positions, across the 4 cancer types. In each cancer type the 100 genes with the highest coverage by hyper/hypomutated regions is shown.
