# Supplementary material for: A Dual Model for Prioritizing Cancer Mutations in the Non-coding Genome Based on Germline and Somatic Events
Source: PLoS Comput Biol. 2015 Nov 20;11(11):e1004583. doi: 10.1371/journal.pcbi.1004583 (PMC4654583; doi:10.1371/journal.pcbi.1004583)
Supplement: S1 Table — (DOCX) [file pcbi.1004583.s008.docx]

**Table S1**. Uniform genomic features used in figures and SNP or SOM models.

| **Name** | **Description** | **Extent (Mb)** | **Reference** | **Model** |
| --- | --- | --- | --- | --- |
| UTR | mRNA untranslated region | 47.23 | Gencode v7[1] | SNP+SOM |
| CDS | Coding sequence | 35.34 | Gencode | SOM |
| Exon.P | Exon of protein coding gene | 91.15 | Gencode | - |
| Intron.P | Intron of protein coding gene | 1236.20 | Gencode | SNP+SOM |
| PCgene | Protein coding gene | 1266.97 | Gencode | SOM |
| lncRNA | Long non-coding RNA | 337.12 | Gencode | SOM |
| Exon.L | Exon of lncRNA | 16.44 | Gencode | - |
| Intron.L | Intron of lncRNA | 324.18 | Gencode | SNP+SOM |
| ncExon | Non coding exon | 30.61 | Gencode | SNP+SOM |
| Intergenic | Intergenic region | 1568.79 | Gencode | SOM |
| 5’SS | 5’splicing site (10bp from the splicing site) | 2.95 | Gencode | - |
| 3’SS | 3’splicing site (50bp from the splicing site) | 13.03 | Gencode | - |
| GC content | Fraction of G or C nucleotide per 1Mb window | - | UCSC [2] | SOM |
| GC H | 1-kb windows with high GC content (GC% > 50) | 308.86 | UCSC | - |
| GC L | 1-kb windows with low GC content (GC% < 30) | 104.89 | UCSC | - |
| Promoter | Promoter | 84.91 | Gencode | SNP+SOM |
| Enhancer | Enhancer | 12.03 | FANTOM5[3] | SNP |
| TFBS | Transcription factor binding site | 947 | ENCODE[4] | SNP |
| cTFBS | Conserved transcription factor binding site | 59.23 | UCSC | SNP+SOM |
| Sensitive | Khurana et al.'s region of high rate of rare SNP | 9.21 | [5] | SOM |
| CR | Conserved region (PhastCons 46 way) | 150.98 | UCSC | SNP+SOM |
| ECS | Evolutionarily conserved RNA structure | 199.68 | [6] | SNP+SOM |
| DNase I | DNase I hypersensitive site (any cell type) | 388.42 | ENCODE | SNP+SOM |
| HE | Highly expressed gene/RNA (RPKM>20) in either cell line | 635.78 | ENCODE | SNP |
| LE | Low expressed gene/RNA (RPKM<0.25) in either cell line | 1002.47 | ENCODE | SNP |
| ER | Early replicated gene/RNA (EL ratio >1) in all cell lines | 418.68 | ENCODE | SNP |
| Recombination rate | Recombination rate averaged per 1Mb window | - | HAPMAP [7] | SOM |
| RR H | 1-kb windows with high recombination rate (> 4.0) | 117.55 | HAPMAP | SNP |
| RR L | 1-kb windows with low recombination rate (< 0.5) | 1034.26 | HAPMAP | SNP |
| GC | G or C base for each nucleotide | - | UCSC | SNP |
