# Supplementary material for: A Dual Model for Prioritizing Cancer Mutations in the Non-coding Genome Based on Germline and Somatic Events
Source: PLoS Comput Biol. 2015 Nov 20;11(11):e1004583. doi: 10.1371/journal.pcbi.1004583 (PMC4654583; doi:10.1371/journal.pcbi.1004583)
Supplement: S2 Table — (DOCX) [file pcbi.1004583.s009.docx]

**Table S2.** Cell-specific genomic features used in figures and SOM models.

|  |  | **Extent (Mb) of feature** | | | |
| --- | --- | --- | --- | --- | --- |
| **Name** | **Description** | Hepg2  (liver) | A549  (lung) | K562  (CLL) | Nhdfad  (melanoma) |
| H3k4me1 | H3k4me1 | 384.12 | 420.25 | 325.92 | 378.14 |
| H3k4me2 | H3k4m2 | 174.18 | 203.29 | 135.32 | 228.49 |
| H3k4me3 | H3k4me3 | 106.66 | 152.19 | 147.31 | 192.53 |
| H3k9ac | H3k9ac | 158.06 | 157.44 | 185.10 | 251.10 |
| H3k9me3 | H3k9me3 | 559.11 | 942.29 | 924.53 | 834.50 |
| H3k27ac | H3k27ac | 130.21 | 174.38 | 146.05 | 353.92 |
| H3k27me3 | H3k27me3 | 767.11 | 861.39 | 641.29 | 695.29 |
| H3k36me3 | H3k36me3 | 511.89 | 705.49 | 499.00 | 611.44 |
| H3k79me2 | H3k79me2 | 314.30 | 430.61 | 269.26 | 354.03 |
| H3K20me1 | H3K20me1 | 605.41 | 753.80 | 772.78 | 499.01 |
| H2az | H2az | 886.95 | 503.30 | 341.67 | 454.64 |
| CTCF | CTCF | 77.77 | 118.31 | 127.48 | 98.23 |
| Ezh2 | Ezh2 | 698.22 | - | 871.32 | 435.09 |
| TFBS | Transcription factor binding site | 286.38 | 164.61 | 348.46 | 65.69 |
| Expression level | RPKM per 1Mb window | - | - | - | - |
| PCgene.HE | Highly expressed protein coding gene (RPKM >20) | 93.08 | 72.36 | 101.08 | 79.21 |
| PCgene.LE | Low expressed protein coding gene (RPKM <0.25) | 422.35 | 222.52 | 457.92 | 311.64 |
| LncRNA.HE | Highly expressed lncRNA (RPKM >20) | 21.34 | 23.70 | 22.49 | 22.08 |
| LncRNA.LE | Low expressed lncRNA (RPKM <0.25) | 165.66 | 91.92 | 176.40 | 125.67 |
|  |  | Hepg2 | Imr90 | K562 | Bg02 |
| Replication time | Replication timing ratio per 1Mb window | - | - | - | - |
| LncRNA.early | Early replicated lncRNA (E/L ratio >1) | 818.79 | 733.59 | 758.60 | 790.78 |
| LncRNA.late | Late replicated lncRNA (E/L ratio <1) | 441.3 | 520.16 | 497.73 | 471.30 |
| PCgene.late | Late replicated protein coding gene (E/L ratio >1) | 140.59 | 142.04 | 132.01 | 125.22 |
| PCgene.early | Early replicated protein coding gene (E/L ratio <1) | 182.39 | 175.87 | 188.71 | 198.26 |
|  |  | Liver hepatocelluar carcinoma | Lung adenocarcinoma | Acute myeloid leukemia | Skin cutaneous melanoma |
| DNA.met H | Average DNA methylation value < 0.4062 | 58.22 | 63.96 | 99.81 | 68.14 |
| DNA.met L | Average DNA methylation value > 0.7245 | 58.22 | 57.26 | 51.85 | 52.38 |
