# Supplementary material for: A Dual Model for Prioritizing Cancer Mutations in the Non-coding Genome Based on Germline and Somatic Events
Source: PLoS Comput Biol. 2015 Nov 20;11(11):e1004583. doi: 10.1371/journal.pcbi.1004583 (PMC4654583; doi:10.1371/journal.pcbi.1004583)
Supplement: S3 Table — (DOCX) [file pcbi.1004583.s010.docx]

**Table S3**. Significance of disease mutation enrichment in high-SNP+low SOM regions, for 4 cancer types.

| **Cancer type** | **Region** | **Region size (nt)** | **HGMD** | **Clivariant** | **P value (1)** |
| --- | --- | --- | --- | --- | --- |
| - | Intergenic | 1568807082 | 913 | 213 |  |
| - | High SNP | 98163148 | 6784 | 1767 |  |
| Liver | Low SOM | 1255672000 | 9719 | 4572 |  |
|  | Low SOM+ high SNP | 56198409 | 5079 | 1393 | <2.2e-16 |
| Lung | Low SOM | 1186445000 | 9714 | 4596 |  |
|  | Low SOM+ high SNP | 56160584 | 5012 | 1391 | <2.2e-16 |
| CLL | Low SOM | 1236512000 | 9580 | 4660 |  |
|  | Low SOM+ high SNP | 56267795 | 4773 | 1332 | <2.2e-16 |
| Melanoma | Low SOM | 1170977000 | 9265 | 4384 |  |
|  | Low SOM+ high SNP | 56148149 | 4892 | 1322 | <2.2e-16 |

(1) P values are computed as follows: disease-associated variants from the HGMD or Clivariant database are counted in high SNP or low SOM *vs*. Low SOM+high SNP regions, along with region sizes, forming a 2x2 matrix for Chi-square test in each cancer type. P values here are statistical significance for both HGMD and Clivariant databases.
