# Supplementary material for: A Dual Model for Prioritizing Cancer Mutations in the Non-coding Genome Based on Germline and Somatic Events
Source: PLoS Comput Biol. 2015 Nov 20;11(11):e1004583. doi: 10.1371/journal.pcbi.1004583 (PMC4654583; doi:10.1371/journal.pcbi.1004583)
Supplement: S4 Table — (DOCX) [file pcbi.1004583.s011.docx]

**Table S4.** Significance of over-enrichment for hypomutated regions within cancer vs non-cancer genes. Enrichment for hypomutated regions was computed as explained in Methods for each independent gene. Then for each gene class (protein-coding, lncRNA, miRNA), a Wilcoxon rank sum test was performed to compare enrichment factors in cancer genes (see Methods for gene lists) and in all genes in the class.

| Gene type | Cancer type | P-value |
| --- | --- | --- |
| Protein-coding  (non-coding parts) | Liver | 1.44E-12 |
|  | Lung | 5.05E-14 |
|  | CLL | 5.22E-15 |
|  | Melanoma | <2.20E-16 |
| lncRNA | Liver | 0.838 |
|  | Lung | 0.158 |
|  | CLL | 0.705 |
|  | Melanoma | 0.903 |
| miRNA | Liver | 0.007 |
|  | Lung | 0.003 |
|  | CLL | 0.011 |
|  | Melanoma | 0.004 |
