# Supplementary material for: A Dual Model for Prioritizing Cancer Mutations in the Non-coding Genome Based on Germline and Somatic Events
Source: PLoS Comput Biol. 2015 Nov 20;11(11):e1004583. doi: 10.1371/journal.pcbi.1004583 (PMC4654583; doi:10.1371/journal.pcbi.1004583)
Supplement: S5 Table — (DOCX) [file pcbi.1004583.s012.docx]

**Table S5**: Biological process GO-term biases (1) in the 100 protein coding genes with highest coverage by hypermutated (high SNP-high SOM) positions (liver cancer and CLL).

| GO biological process complete | # | # | expected | Fold Enrichment | +/- | P value (2) |
| --- | --- | --- | --- | --- | --- | --- |
| Liver cancer | | | | | | |
| Unclassified | 4272 | 17 | 18.88 | .90 | - | 0.00E00 |
| transcription from RNA polymerase II promoter | 781 | 19 | 3.45 | > 5 | + | 1.04E-05 |
| gene expression | 3825 | 41 | 16.91 | 2.43 | + | 5.55E-05 |
| cellular nitrogen compound metabolic process | 5112 | 48 | 22.60 | 2.12 | + | 9.12E-05 |
| nucleobase-containing compound metabolic process | 4372 | 43 | 19.32 | 2.23 | + | 2.58E-04 |
| RNA metabolic process | 3373 | 37 | 14.91 | 2.48 | + | 2.61E-04 |
| nucleic acid metabolic process | 3874 | 40 | 17.12 | 2.34 | + | 2.85E-04 |
| cellular nitrogen compound biosynthetic process | 3407 | 37 | 15.06 | 2.46 | + | 3.42E-04 |
| nucleobase-containing compound biosynthetic process | 2962 | 34 | 13.09 | 2.60 | + | 4.21E-04 |
| RNA biosynthetic process | 2680 | 32 | 11.85 | 2.70 | + | 5.01E-04 |
| transcription, DNA-templated | 2560 | 31 | 11.32 | 2.74 | + | 6.41E-04 |
| nucleic acid-templated transcription | 2561 | 31 | 11.32 | 2.74 | + | 6.47E-04 |
| heterocycle biosynthetic process | 3043 | 34 | 13.45 | 2.53 | + | 8.19E-04 |
| aromatic compound biosynthetic process | 3044 | 34 | 13.45 | 2.53 | + | 8.25E-04 |
| nitrogen compound metabolic process | 5475 | 48 | 24.20 | 1.98 | + | 9.12E-04 |
| Chronic lymphocytic leukemia (CLL) | | | | | | |
| positive regulation of transcription from RNA polymerase II promoter | 987 | 17 | 4.46 | 3.81 | + | 1.52E-02 |

(1) Amigo V1.8: http://amigo1.geneontology.org

(2) Bonferroni-adjusted P-value
