# Supplementary material for: A Dual Model for Prioritizing Cancer Mutations in the Non-coding Genome Based on Germline and Somatic Events
Source: PLoS Comput Biol. 2015 Nov 20;11(11):e1004583. doi: 10.1371/journal.pcbi.1004583 (PMC4654583; doi:10.1371/journal.pcbi.1004583)
Supplement: S6 Table — (DOCX) [file pcbi.1004583.s013.docx]

**Table S6**. Mammalian long non-coding RNAs experimentally shown to be associated with different cancer types from a literature search.

| Chromosome | Start | End | LncRNA | Size(bp) | Reference |
| --- | --- | --- | --- | --- | --- |
| chr9 | 21994789 | 22029563 | ANRIL | 503 | [8] |
| chr1 | 173833038 | 173837125 | GAS5 | 632 | [9] |
| chr12 | 54356095 | 54362515 | HOTAIR | 2337 | [10] |
| chr7 | 27135712 | 27139585 | HOTAIRM1 | 483 | [11] |
| chr6 | 8652441 | 8654459 | HULC | 500 | [12] |
| chr3 | 116428634 | 116435887 | LOC285194 | 2105 | [13] |
| chr3 | 50137035 | 50138421 | LUST | 1386 | [14] |
| chr6 | 136265388 | 136282959 | NTT | 17572 | [15] |
| chr9 | 79379353 | 79402465 | PCA3 | 3735 | [16] |
| chr8 | 128025398 | 128033259 | PCAT1 | 1992 | [17] |
| chr2 | 193614570 | 193641625 | PCGEM1 | 1590 | [18] |
| chr9 | 33673501 | 33677418 | PTENP1 | 3932 | [19] |
| chr3 | 181417385 | 181433076 | Sox2ot | 2970 | [20] |
| chr5 | 139929652 | 139937678 | SRA | 1965 | [21] |
| chr22 | 31365633 | 31375381 | TUG1 | 7105 | [22] |
| chr19 | 15939756 | 15946230 | UCA1 | 1413 | [23] |
| chrX | 73040494 | 73072588 | XIST | 19271 | [24] |
| chr8 | 128092118 | 128104845 | PRNCR1 | 12756 | [18] |
| chr14 | 101292444 | 101327363 | MEG3 | 1855 | [25] |
| chr11 | 2016405 | 2019065 | H19 | 2308 | [26-27] |
| chr11 | 65265232 | 65273940 | MALAT1 | 8708 | [28-29] |
| chr14 | 61283510 | 61285560 | HIF1A-AS2 | 2050 | [30] |
| chr17 | 23111183 | 23134213 | Anti-NOS2A | 23 | [31] |
| chr7 | 148315552 | 148317449 | GHET1 | 1898 | [32] |
| chr20 | 5048232 | 5048615 | PCNA-AS1 | 384 | [33] |

**References for Supplementary Tables**

1. Harrow J, Frankish A, Gonzalez JM, Tapanari E, Diekhans M, Kokocinski F, et al. GENCODE: The reference human genome annotation for the ENCODE project. Genome Res. 2012;22: 1760–1774. doi:10.1101/gr.135350.111

2. Karolchik D, Barber GP, Casper J, Clawson H, Cline MS, Diekhans M, et al. The UCSC Genome Browser database: 2014 update. Nucleic Acids Res. 2014;42: 764–770. doi:10.1093/nar/gkt1168

3. Andersson R, Gebhard C, Miguel-Escalada I, Hoof I, Bornholdt J, Boyd M, et al. An atlas of active enhancers across human cell types and tissues. Nature. 2014;507: 455–61. doi:10.1038/nature12787

4. Rosenbloom KR, Sloan C a., Malladi VS, Dreszer TR, Learned K, Kirkup VM, et al. ENCODE Data in the UCSC Genome Browser: Year 5 update. Nucleic Acids Res. 2013;41: 56–63. doi:10.1093/nar/gks1172

5. Khurana E, Fu Y, Colonna V, Mu XJ, Kang HM, Lappalainen T, et al. Integrative annotation of variants from 1092 humans: application to cancer genomics. Science. 2013;342: 1235587. doi:10.1126/science.1235587

6. Smith M a., Gesell T, Stadler PF, Mattick JS. Widespread purifying selection on RNA structure in mammals. Nucleic Acids Res. 2013;41: 8220–8236. doi:10.1093/nar/gkt596

7. Altshuler DM, Gibbs R a, Peltonen L, Altshuler DM, Gibbs R a, Peltonen L, et al. Integrating common and rare genetic variation in diverse human populations. Nature. 2010;467: 52–58. doi:10.1038/nature09298

8. Kotake Y, Nakagawa T, Kitagawa K, Suzuki S, Liu N, Kitagawa M, et al. Long non-coding RNA ANRIL is required for the PRC2 recruitment to and silencing of p15(INK4B) tumor suppressor gene. Oncogene. Nature Publishing Group; 2011;30: 1956–1962. doi:10.1038/onc.2010.568

9. Sun M, Jin F, Xia R, Kong R, Li J, Xu T, et al. Decreased expression of long noncoding RNA GAS5 indicates a poor prognosis and promotes cell proliferation in gastric cancer. BMC Cancer. 2014;14: 319. doi:10.1186/1471-2407-14-319

10. Gupta R a, Shah N, Wang KC, Kim J, Horlings HM, Wong DJ, et al. Long non-coding RNA HOTAIR reprograms chromatin state to promote cancer metastasis. Nature. Nature Publishing Group; 2010;464: 1071–1076. doi:10.1038/nature08975

11. Zhang X, Weissman SM, Newburger PE. Long intergenic non-coding RNA HOTAIRM1 regulates cell cycle progression during myeloid maturation in NB4 human promyelocytic leukemia cells. RNA Biol. 2014;11: 1–11. doi:10.4161/rna.28828

12. Panzitt K, Tschernatsch MMO, Guelly C, Moustafa T, Stradner M, Strohmaier HM, et al. Characterization of HULC, a Novel Gene With Striking Up-Regulation in Hepatocellular Carcinoma, as Noncoding RNA. Gastroenterology. 2007;132: 330–342. doi:10.1053/j.gastro.2006.08.026

13. Liu Q, Huang J, Zhou N, Zhang Z, Zhang A, Lu Z, et al. LncRNA loc285194 is a p53-regulated tumor suppressor. Nucleic Acids Res. 2013;41: 4976–4987. doi:10.1093/nar/gkt182

14. Rintala-Maki ND, Sutherland LC. Identification and characterisation of a novel antisense non-coding RNA from the RBM5 gene locus. Gene. Elsevier B.V.; 2009;445: 7–16. doi:10.1016/j.gene.2009.06.009

15. Delgado André N, De Lucca FL. Non-coding transcript in T cells (NTT): Antisense transcript activates PKR and NF-κB in human lymphocytes. Blood Cells, Mol Dis. 2008;40: 227–232. doi:10.1016/j.bcmd.2007.08.005

16. Gezer U, Ph D, Tiryakioglu D, Sc M, Bilgin E, Sc M, et al. Androgen Stimulation of PCA3 and miR-141 and Their Release from Prostate Cancer Cells. 2015;16: 488–493.

17. Prensner JR, Iyer MK, Balbin OA, Dhanasekaran SM, Cao Q, Brenner JC, et al. Transcriptome sequencing across a prostate cancer cohort identifies PCAT-1, an unannotated lincRNA implicated in disease progression. Nat Biotechnol. Nature Publishing Group; 2011;29: 742–749. doi:10.1038/nbt.1914

18. Yang L, Lin C, Jin C, Yang JC, Tanasa B, Li W, et al. lncRNA-dependent mechanisms of androgen-receptor-regulated gene activation programs. Nature. Nature Publishing Group; 2013;500: 598–602. doi:10.1038/nature12451

19. Chen C-L, Tseng Y-W, Wu J-C, Chen G-Y, Lin K-C, Hwang S-M, et al. Suppression of hepatocellular carcinoma by baculovirus-mediated expression of long non-coding RNA PTENP1 and MicroRNA regulation. Biomaterials. Elsevier Ltd; 2015;44: 71–81. doi:10.1016/j.biomaterials.2014.12.023

20. Askarian-Amiri ME, Seyfoddin V, Smart CE, Wang J, Kim JE, Hansji H, et al. Emerging role of long non-coding RNA SOX2OT in SOX2 regulation in breast cancer. PLoS One. 2014;9: 1–10. doi:10.1371/journal.pone.0102140

21. Leygue E, Dotzlaw H, Watson PH, Murphy LC. Expression of the Steroid Receptor RNA Activator in Human Breast Tumors Advances in Brief Expression of the Steroid Receptor RNA Activator in Human Breast Tumors 1. Cancer Res. 1999;59: 4190–4193.

22. Zhang E, Yin D, Sun M, Kong R, Liu X, You L, et al. P53-regulated long non-coding RNA TUG1 affects cell proliferation in human non-small cell lung cancer, partly through epigenetically regulating HOXB7 expression. Cell Death Dis. 2014;5: e1243. doi:10.1038/cddis.2014.201

23. Yang C, Li X, Wang Y, Zhao L, Chen W. Long non-coding RNA UCA1 regulated cell cycle distribution via CREB through PI3-K dependent pathway in bladder carcinoma cells. Gene. 2012;496: 8–16. doi:10.1016/j.gene.2012.01.012

24. McHugh C a., Chen C-K, Chow A, Surka CF, Tran C, McDonel P, et al. The Xist lncRNA interacts directly with SHARP to silence transcription through HDAC3. Nature. 2015; doi:10.1038/nature14443

25. Benetatos L, Vartholomatos G, Hatzimichael E. MEG3 imprinted gene contribution in tumorigenesis. Int J Cancer. 2011;129: 773–779. doi:10.1002/ijc.26052

26. Fellig Y, Ariel I, Ohana P, Schachter P, Sinelnikov I, Birman T, et al. H19 expression in hepatic metastases from a range of human carcinomas. J Clin Pathol. 2005;58: 1064–1068. doi:10.1136/jcp.2004.023648

27. Tsang WP, Ng EKO, Ng SSM, Jin H, Yu J, Sung JJY, et al. Oncofetal H19-derived miR-675 regulates tumor suppressor RB in human colorectal cancer. Carcinogenesis. 2010;31: 350–358. doi:10.1093/carcin/bgp181

28. Hirata H, Hinoda Y, Shahryari V, Deng G, Nakajima K, Tabatabai ZL, et al. Long Noncoding RNA MALAT1 Promotes Aggressive Renal Cell Carcinoma through Ezh2 and Interacts with miR-205. Cancer Res. 2015;75: 1322–1331. doi:10.1158/0008-5472.CAN-14-2931

29. Gutschner T, Hämmerle M, Eißmann M, Hsu J, Kim Y, Hung G, et al. The noncoding RNA MALAT1 is a critical regulator of the metastasis phenotype of lung cancer cells. Cancer Res. 2013;73: 1180–1189. doi:10.1158/0008-5472.CAN-12-2850

30. Chen W, Huang M, Kong R, Xu T, Zhang E, Xia R, et al. Antisense Long Noncoding RNA HIF1A-AS2 Is Upregulated in Gastric Cancer and Associated with Poor Prognosis. Dig Dis Sci. 2015; doi:10.1007/s10620-015-3524-0

31. Korneev S a, Korneeva EI, Lagarkova M a, Kiselev SL, Critchley G, O’Shea M. Novel noncoding antisense RNA transcribed from human anti-NOS2A locus is differentially regulated during neuronal differentiation of embryonic stem cells. RNA. 2008;14: 2030–2037. doi:10.1261/rna.1084308

32. Yang F, Xue X, Zheng L, Bi J, Zhou Y, Zhi K, et al. Long non-coding RNA GHET1 promotes gastric carcinoma cell proliferation by increasing c-Myc mRNA stability. FEBS J. 2014;281: 802–813. doi:10.1111/febs.12625

33. Yuan S-X, Tao Q-F, Wang J, Yang F, Liu L, Wang L-L, et al. Antisense long non-coding RNA PCNA-AS1 promotes tumor growth by regulating proliferating cell nuclear antigen in hepatocellular carcinoma. Cancer Lett. Elsevier Ireland Ltd; 2014;349: 87–94. doi:10.1016/j.canlet.2014.03.029
