# Supplementary material for: A Dual Model for Prioritizing Cancer Mutations in the Non-coding Genome Based on Germline and Somatic Events
Source: PLoS Comput Biol. 2015 Nov 20;11(11):e1004583. doi: 10.1371/journal.pcbi.1004583 (PMC4654583; doi:10.1371/journal.pcbi.1004583)
Supplement: S1 Text — (DOCX) [file pcbi.1004583.s015.docx]

## Supplemental Methods

Calibration, feature selection and validation of random forest models

## Random Forest Models

The random forests (RF) approach involves producing multiple regression trees, which are then combined to make a single consensus prediction for a given observation (Breiman L, 2001).

We generated the SNP RF model and the SOM RF model using the *randomForest* R package. The RF model is composed of an aggregate collection of regression trees, each created from boostrapped training samples: each branch is selected from a random subset of a given number (denoted be *mtry*) of the input variables (data columns). The two main parameters are *mtry* and *ntree,* the number of trees in the forest. We used the mean squared error (abbreviated MSE) as a measure of the prediction accuracy of the RF model. Two MSE error estimates are used in the validation procedure: the OOB error and the cross-validation error. An important feature of RFs is its use of *out-of-bag* (OOB) samples. An OOB sample is the set of observations which are not used for building the current tree, and can be used to estimate the MSE error; it can be shown that an OOB error estimate is almost identical to that obtained by K-fold cross-validation.

RF models have the advantage of giving a summary of the importance of each variable based on the randomized variable selection process used to grow the RF. An estimation of variable importance is provided by *IncNodePurity*, which measures the decrease in tree node purity that results from all splits of a given variable over all trees. This measure can be used to rank variables by the strength of their relation to the response variable, for interpretation purposes.

## Model Calibration

We first tuned the two parameters *mtry* and *ntree* of the RF method. Figure 1 shows the OOB error progression on 500 trees for random forests using different parameters *mtry*. MSE errors stabilize at about 400 trees, so we see that *ntree*=500 (default value) was sufficient to give good performance for the SNP model and for the SOM model.

In a regression framework, the default value of *mtry* is [p/3] where p is the number of variables. The case *mtry*=p corresponds to bagging (or bootstrap aggregation), a general purpose procedure for reducing the variance of a statistical learning method. For the SNP data we have p=18 and the default value of *mtry* is 5. Note that a larger *mtry* is best suited to the SNP and SOM data, according to the MSE error (Figure 1 and Figure 4). We considered the gain in MSE error was small enough for mtry greater than 7 for the SNP model and 10 for the liver cancer SOM model.

Assessment of variable importance is performed using IncNodePurity, with larger values indicating more important variables. We examined the RF variable importances behavior for different values of *ntree* and *mtry*. In Figure 2 and Figure 5, a graphical representation of the variable importances is shown using 3 values of mtry (5 the default, 7 and 14 for SNP model, 10, 20 and 30 for SOM model of liver cancer) and two values of *ntree* (the 500 default and 1000). The magnitude of the variable importances is increased with larger values of *mtry*, but we get nearly the same order for all variables in every run of the procedure and with every value of *mtry*. Moreover, using a small value of *mtry* is preferred in the presence of correlated predictors. We chose mtry=7 for the SNP model and mtry=10 for the SOM models of liver cancer, lung cancer, CLL and melanoma, respectively, based on lower MSE errors and smaller mtry values (Fig 1 and 4).

## Feature selection

We used the R *VSURF* package to perform variable selection. The selection procedure is based on a ranking of the explanatory variables using the random forests score of importance and a stepwise ascending strategy (Genuer 2010). The first step eliminates the noisy variables and the second step selects the variables leading to the smallest OOB error. One advantage in using the VSURF procedure lies in its robustness with respect to the choice of *mtry* and *ntree*.

## Model Validation

RFs were grown with ntree=500 for all models. We used mtry=7 for the SNP model and mtry=10 for the SOM models. The SNP RF model was trained using 16 explanatory variables. The SOM RF models were estimated using 21, 22, 29 and 23 explanatory variables selected by VSURF for liver cancer, lung cancer, CLL and melanoma respectively. The validation of the two models is given in terms of MSE. We used 10-fold cross-validation to compute the prediction error.

We compared the prediction error of the RF model to the prediction error obtained training a multiple regression linear model with the same input variables involved. We see that RFs outperform a linear model for the SNP and cancer mutation data (Figure 3A and Figure 6A).


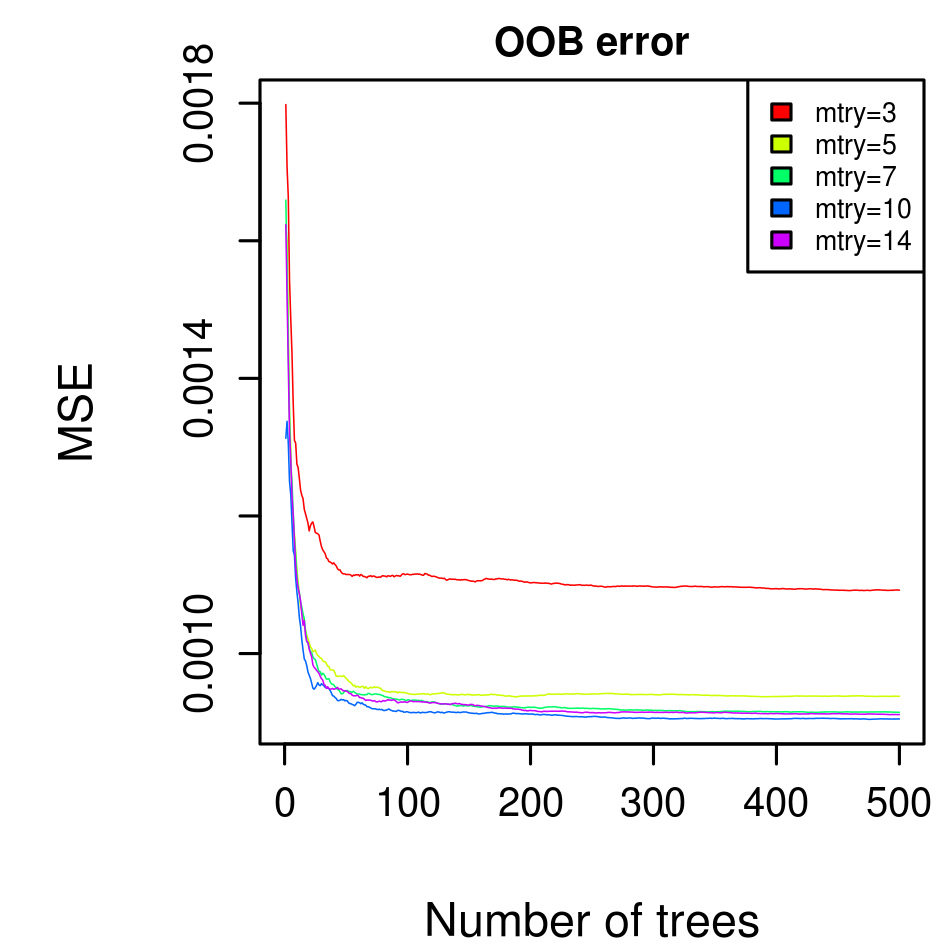


Figure 1: MSE sensitivity to ntree and mtry (SNP model)


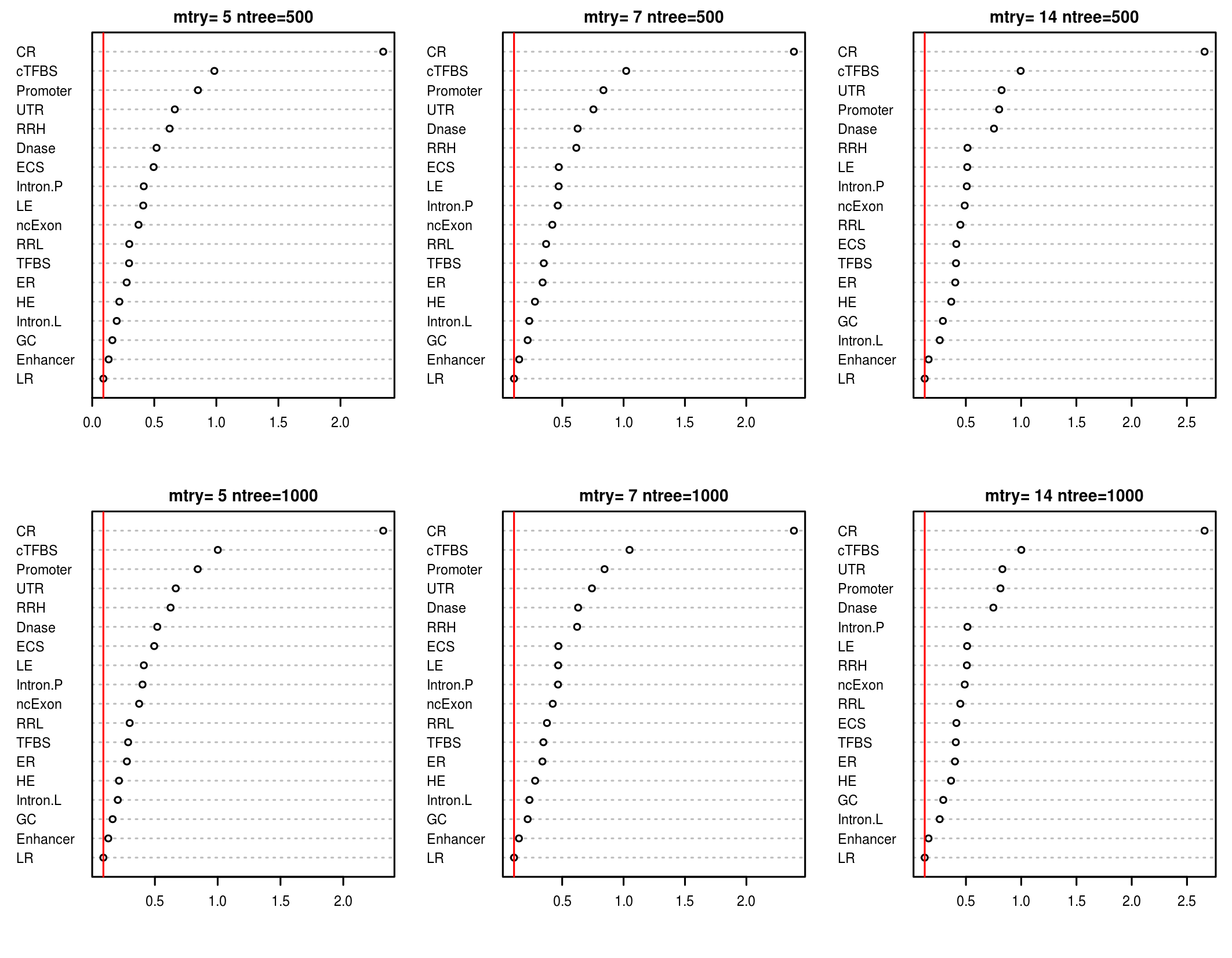
Figure 2: variable importance (*lncNodePurity*) sensitivity to ntree and mtry (red line: absolute value of minimum importance among all features in the SNP RF model)


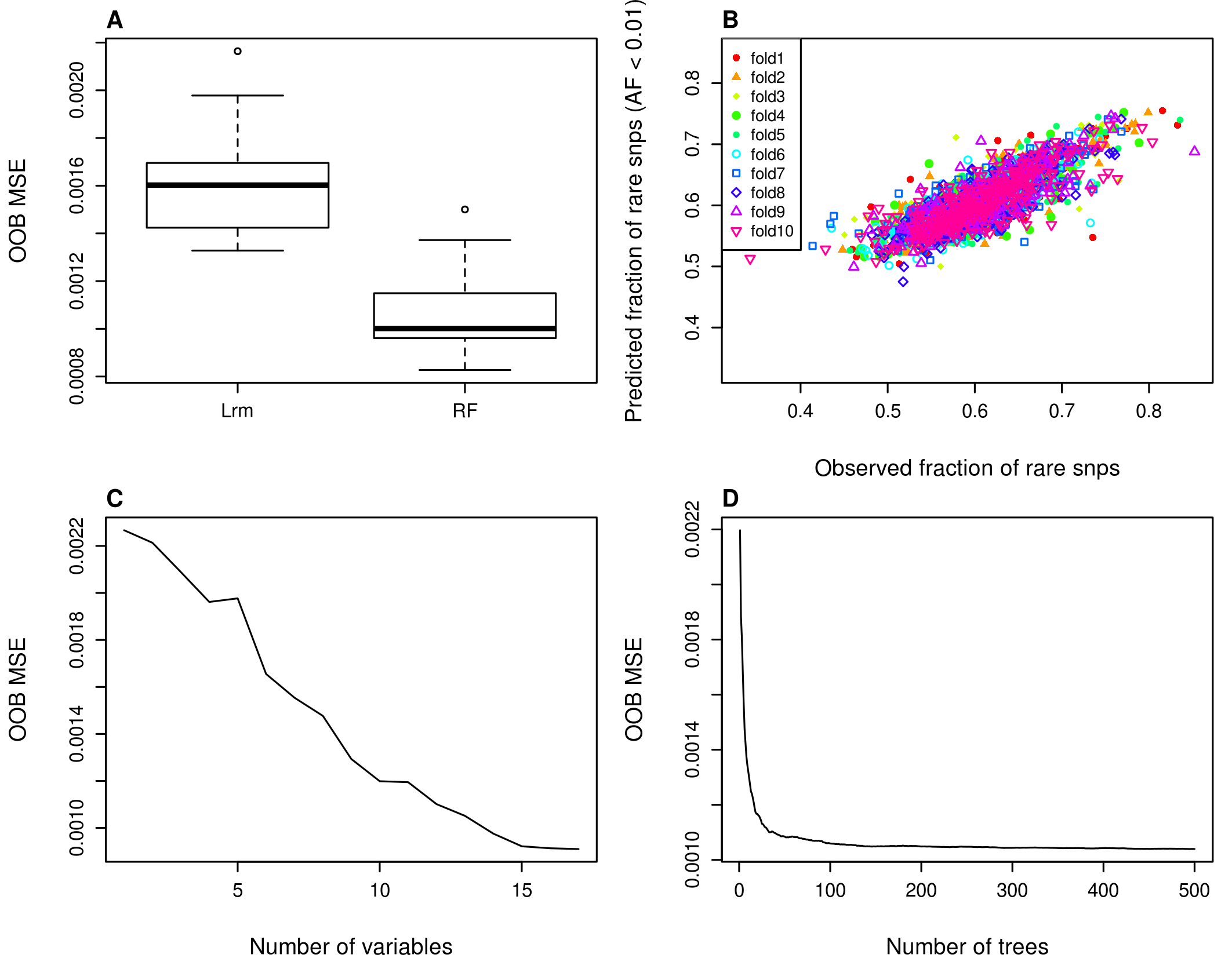


Figure 3: Validation of the SNP model. **A.** MSE for linear regression model (Lrm) and Random forest (RF) with 10-fold cross validation (SNP model); **B**. observed and predicted fraction of rare SNPs (AF < 0.01) with 10-fold cross validation; **C**. the number of variables remained in the RF model minimizes the OOB error; **D**. the default number of trees in the RF model minimizes the OOB error.


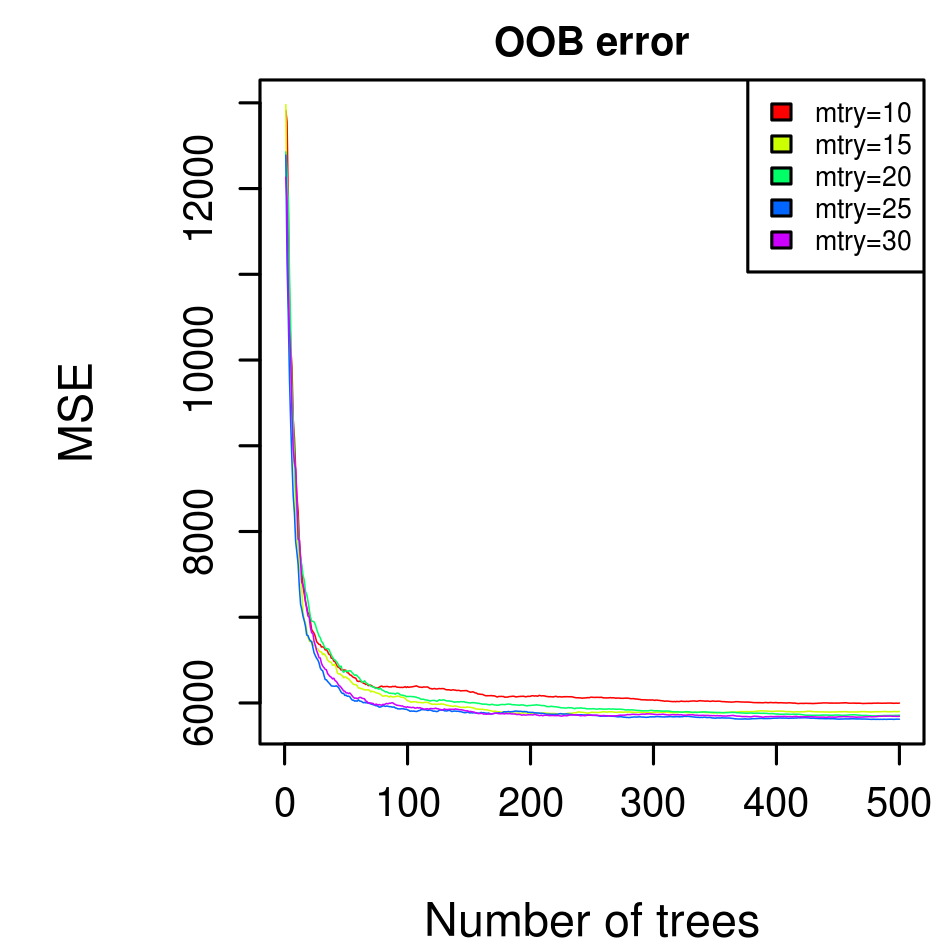


Figure 4 : MSE sensitivity to ntree and mtry (SOM liver cancer model)


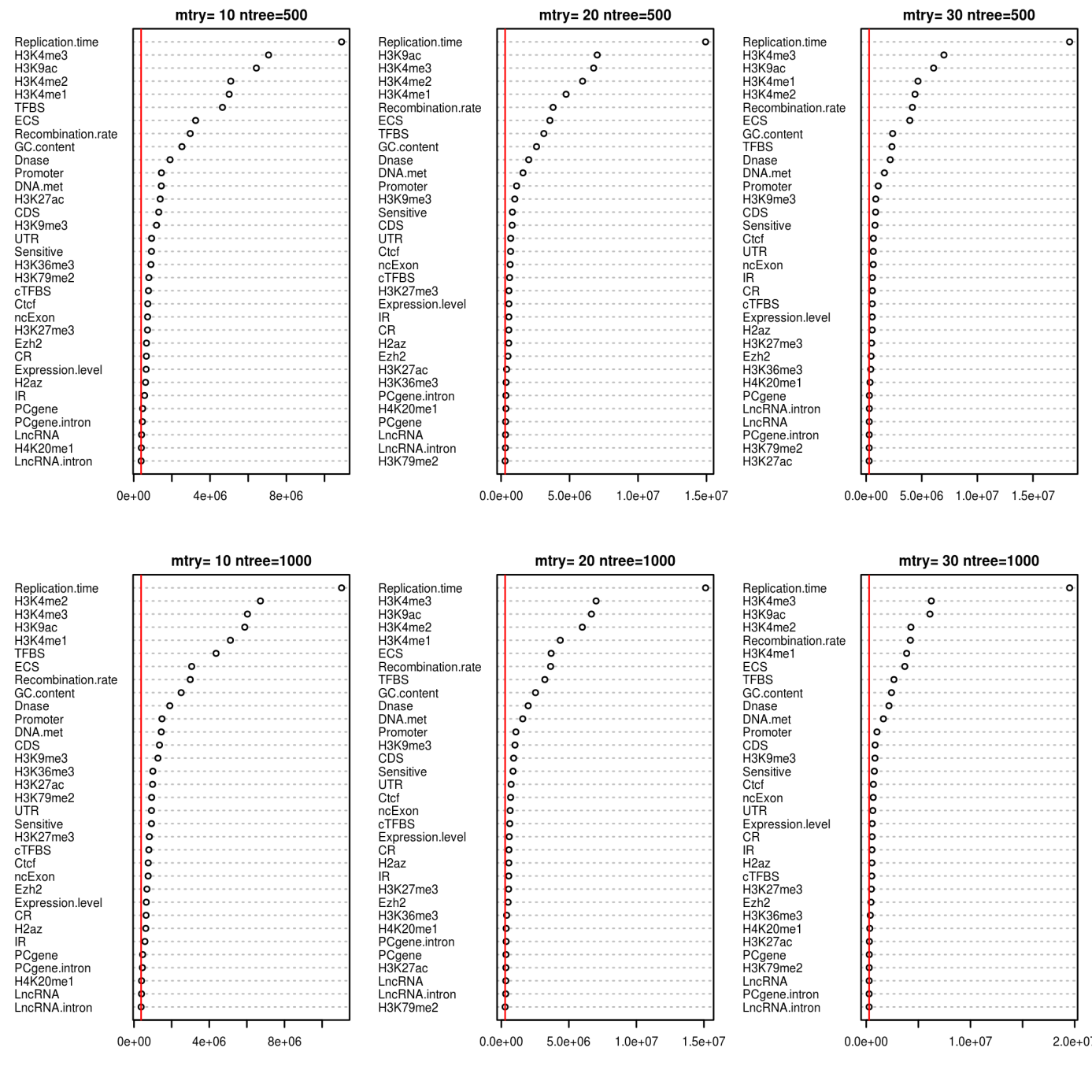


Figure 5: variable importance (lncNodePurity) sensitivity to ntree and mtry (red line: the absolute value of minimum importance among all features in the SOM model of liver cancer)


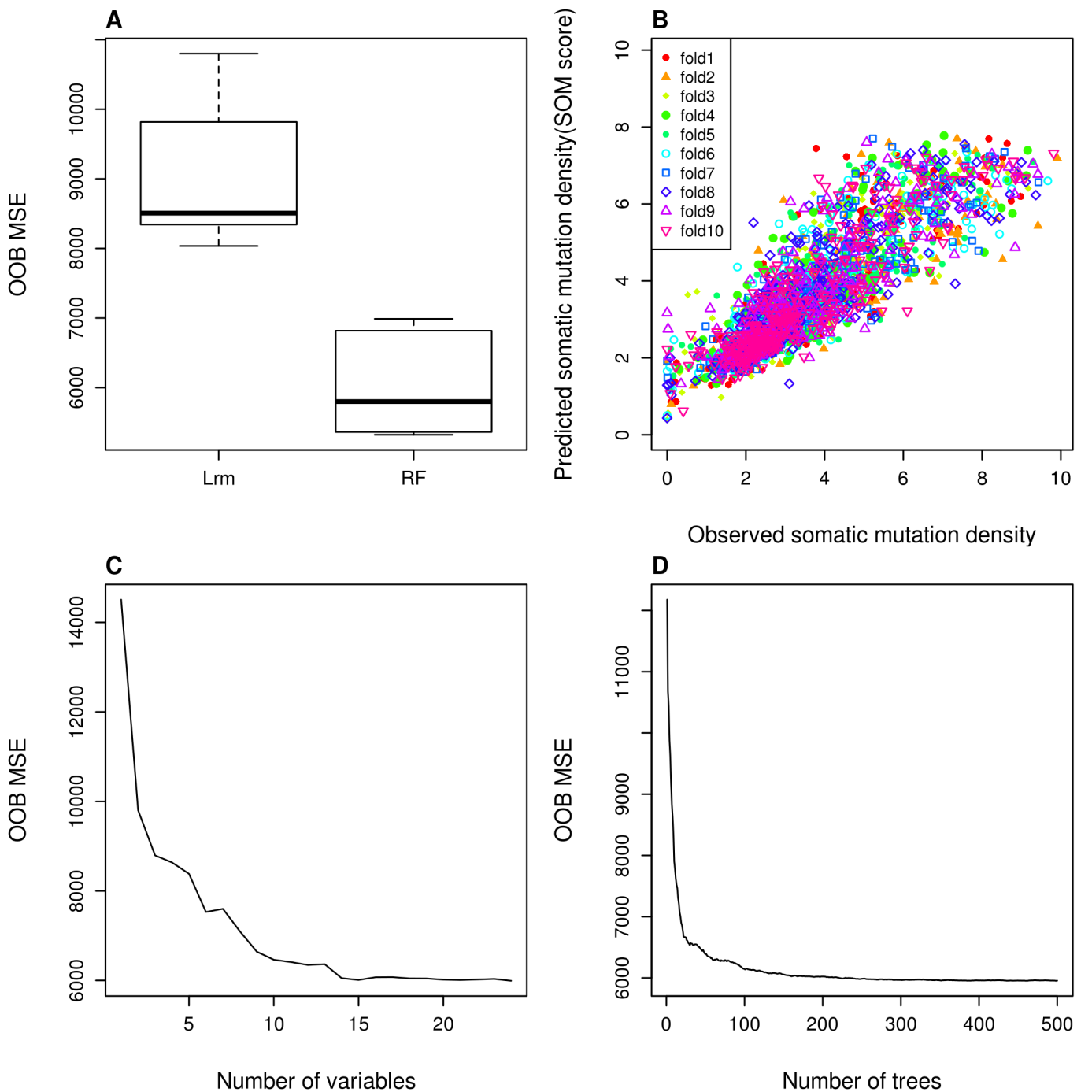


Figure 6: Validation of the liver cancer SOM model. **A**.MSE for linear regression model (Lrm) and Random forest (RF) with 10-fold cross validation (SOM model); B. observed and predicted somatic mutation density divided by 88 patients with 10-fold cross validation; **C**. the number of variables remained in the RF model minimizes the OOB error; **D.** the number of trees in the RF model minimizing the OOB error.

**Data files:**

SNP_model_training_set.tsv

Training set for SNP model. Contains 44130 rows and 23 columns, each row represents one combination of features through out the non-coding human genome.

Columns 1-23 are: Rare_snp (the number of rare snps for each combination), Neutral_snp (the number of neutral snps for each combination), Clivariant (the number of CliVar disease-associated variants for each combination), HGMD (the number of HGMD disease-associated variants for each combination), Size (the number of nucleotides for each combination) and 18 binary feature vectors.

Human_genome_liver_cancer_1Mbwindow_training_set.tsv

Training set for SOM model of liver cancer. Contains 2846 rows and 34 columns, each row represents one non-overlapping 1-Mb window.

Columns 1-34 are: Liver_mutation(the number of liver cancer mutations for each window) and 33 feature vectors with continuous values.

Human_genome_som_liver_cancer_annotation.tsv

Annotation data of liver cancer. Contains 2832686 rows and 36 columns. Each row represents one 1-Mb window. The 1Mb-window was slided across the human genome with a step size of 1Kb, forming 2832686 row annotations. The first three columns are the genetic coordinate for each 1-Mb window, followed by 33 feature vectors.

RFmodel_workflow.R

R scripts used for training, feature selection and prediction of the SNP and SOM models.

RF_SNP_validation.R

RF_SOM_validation.R

R scripts for model calibration, feature selection and validation of the SNP and SOM models.
